# Delayed blastocyst development is associated with altered metabolism and proteome in male and female bovine embryos^†^

**DOI:** 10.1093/biolre/ioaf058

**Published:** 2025-03-21

**Authors:** Kyle J Fresa, Ming-Hao Cheng, Keira Y Larson, Alexandra A Crook, Anthony J Saviola, Raul A Gonzalez-Castro, Thomas W Chen, Elaine M Carnevale

**Affiliations:** Department of Biomedical Sciences, Colorado State University, Fort Collins, Colorado, USA; Department of Electrical and Computer Engineering, Colorado State University, Fort Collins, Colorado, USA; University of Colorado School of Medicine Proteomics Core, Aurora, Colorado, USA; University of Colorado School of Medicine Proteomics Core, Aurora, Colorado, USA; University of Colorado School of Medicine Proteomics Core, Aurora, Colorado, USA; Department of Biomedical Sciences, Colorado State University, Fort Collins, Colorado, USA; Department of Electrical and Computer Engineering, Colorado State University, Fort Collins, Colorado, USA; School of Biomedical Engineering, Colorado State University, Fort Collins, Colorado, USA; Department of Biomedical Sciences, Colorado State University, Fort Collins, Colorado, USA

**Keywords:** embryo, bovine, metabolism, delayed, proteome, oxidative, mitochondria, biosynthesis, catabolic, anabolic, blastocyst

## Abstract

Developmentally delayed embryos are associated with reduced implantation potential and live birth rates; however, inherent causes of delayed development are not well understood. Metabolism during preimplantation development is responsible for the production of energy and biosynthetic material to support growth, and disturbances to these pathways can reduce embryo viability. The present study utilized electrochemical microsensors to determine differences in rates for oxygen consumption, extracellular acidification, and hydrogen peroxide production between normal and slow-growing, male and female bovine blastocysts. In addition, pooled samples of blastocysts were subjected to proteomic analysis to determine differences in the abundance of proteins associated with metabolism between the sexes and developmental timing status. In comparison to blastocysts developing over a normal timespan, blastocysts forming 1–2 days later had a higher oxygen consumption rate, differences in abundance of electron transport complex proteins, and reduced abundance of biosynthetic enzymes when compared to blastocysts developing during a normal timeline. Embryo sex resulted in unique differences in metabolic enzyme abundance with potentially different contributions to delayed development. In addition, male and female blastocysts had differential protein abundances, indicating differences in metabolic pathway activity. Therefore, embryos that took longer to reach the blastocyst stage of development appeared to have an imbalance between energy production and biosynthetic activity, which could differentially impact male and female embryos.

## Teaser

Delayed blastocyst development is associated with greater oxidative metabolism and less abundant proteins involved with biosynthesis, suggesting an imbalance of catabolic and anabolic activity required for survival.

## Graphical Abstract

**Graphical Abstract:**
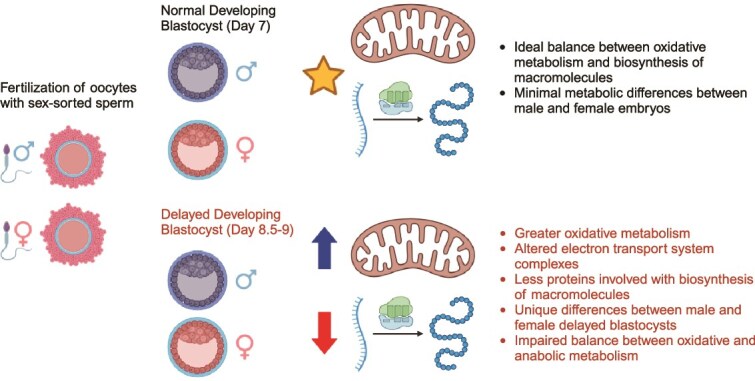

## Introduction

Preimplantation embryo development is characterized by rapid growth that is critical for pregnancy establishment in the cow [1]. Following fertilization, mammalian embryos must undergo cleavage, compaction into a morula, and formation of a blastocyst containing an inner cell mass, trophectoderm, and blastocoel to be considered viable [2]. Blastocysts, the most commonly used developmental stage for embryo transfer and biopsy, usually form after fertilization from 6 to 7 days in cows, 5 to 6 days in humans, and 4 to 5 days in mice; however, a percentage of embryos require additional time to develop to the blastocyst stage [3–5]. The causes of delayed embryo development are not well understood, although studies have correlated maternal and embryo intrinsic factors, such as maternal aging and aneuploidy, to slower embryo growth [3, 6]. Slower-developing embryos are often less viable in the bovine [7], and transfer of in vitro–produced, slow-growing embryos are associated with lower implantation, clinical pregnancy, and live birth rates in humans [8, 9], although the reasons behind this are not yet fully understood. Therefore, understanding the cause of delayed embryo development is essential to determine if culture methods or treatments can be developed to improve the competence of embryos with delayed developmental timing.

During embryo development, metabolic pathways in the cytosol and mitochondria provide the necessary energy and biosynthetic material for adenosine triphosphate (ATP) production, synthesis of macromolecules, and cell proliferation [10]. Bovine embryos are highly prolific from the blastocyst to the conceptus stage, doubling in length every day between days 9 and 16 [1, 11]. To sustain the rapid cell proliferation required for embryo survival during this period, a balance of anabolic and catabolic activities is necessary [12]. The electron transport system, consisting of several protein complexes, forms a proton gradient in the mitochondria that drives the most ATP production in embryos [13]. In addition, adequate amounts of metabolic intermediates must be reserved for macromolecule biosynthesis to support growth [10, 14]. Theories such as the quiet embryo hypothesis have historically suggested that less mitochondrial activity is associated with improved developmental potential, allowing for more emphasis on anabolic activity [15]. In contrast, metabolic activity that is too low has also been shown to be detrimental to efficient embryo growth [16]. Environmental conditions, including culture conditions such as temperature and pH, can also affect the rate of embryo growth [5, 17, 18]. However, why certain embryos take longer to develop than others under the same culture conditions is not clear and is potentially associated with intrinsic embryo differences.

An optimal zone likely exists between low and high metabolic activity that promotes an ideal balance between catabolic and anabolic activity [19]. Few studies have accurately identified the extent of these margins, and studies have been limited to indirect estimates of embryo metabolism, such as spent media metabolite concentrations. However, metabolic activity is dynamic during embryo development, including various changes in substrate metabolism and expression of metabolic enzymes at the blastocyst stage [20]. Our previous work utilized novel electrochemical microsensors to quantify metabolic activities in oocytes and embryos derived from cows and horses and associated with developmental stage and maternal conditions [21–24]. The use of these sensors offers a comprehensive assessment of metabolic activities of individual embryos, providing insight into mitochondrial function, glycolysis, and oxidative stress. Direct measurements of embryo metabolic parameters could provide a novel method to identify alterations in individual embryo metabolic status, allowing new insight into why some embryos are delayed in developmental timing.

In the present study, we investigated metabolic activity associated with normal and delayed blastocyst formation of male and female embryos to better understand intrinsic embryo factors that affect developmental timing and associated viability. We postulated that blastocysts forming over a delayed timeline have intrinsic defects and compensatory mechanisms associated with altered metabolic function, which can affect anabolic versus catabolic activity and may differ by genetic sex. To test this hypothesis, we used novel microsensors to directly assess individual embryo metabolic parameters, including measurements of oxygen consumption rate (OCR), extracellular acidification rate (ECAR), and reactive oxygen species (ROS) production. The proteome of male or female embryos, considered to be developing on a normal or delayed timeline, was assessed for differential catabolic, antioxidant, and biosynthetic proteins. The association of embryo metabolism and proteome were compared for normal- or delayed-developing embryos identified as male or female.

## Methods

### Experimental design

In vitro matured, bovine cumulus–oocyte complexes (COCs) were fertilized with male- or female-sexed sperm. Seven days after the start of oocyte/sperm coincubation, blastocysts (defined as all embryos with the blastocoel >50% of the embryo proper for this study) were removed from culture and considered normal in developmental timing (Normal). Approximately 1.5–2 days later, the remaining embryos that formed into blastocysts were removed from culture and considered delayed in developmental timing (Delayed). Male and female embryos considered Normal or Delayed were placed in a microchamber for the analysis of OCR, ECAR, and hydrogen peroxide production rate, indicative of ROS production. Additional male and female blastocysts considered Normal or Delayed were pooled for proteomic analyses (Supplemental Figure S1).

### Embryo culture

All investigations were conducted in accordance with the relevant institutional and/or national guidelines and standards. Cumulus-oocyte complexes were harvested from slaughterhouse-derived ovaries and selected based on having a compact cumulus mass of more than or equal to three layers. Chemically defined media formulated for bovine oocyte maturation (CDM-M), fertilization (CDM-F), two-step embryo culture (CDM1 and CDM2), and 4-(2-hydroxyethyl)-1-piperazineethanesulfonic acid (HEPES)-buffered holding media for room atmosphere (H-CDM) were made in-house at the Animal Reproduction and Biotechnology Laboratory at Colorado State University based on previously published and modified formulas [25, 26]. Fatty acid–free bovine serum albumin (BSA) was used in media formulations (Sigma, St. Louis, MO).

Oocyte maturation, fertilization, and embryo culture were performed in four-well dishes (NUNC, Thermo Fisher, Waltham, MA) with 0.5 ml of medium per well and 30–40 oocytes or 20–30 embryos per well. Approximately 300 COCs per replicate were matured in CDM-M, with additions of 0.1 mM cysteamine, 1 μg/μL estradiol 17β, 1 μg/μL luteinizing hormone (LH, USDA-LH-B-5, United States Department of Agriculture), 15 ng/μL follicle-stimulating hormone, (FSH, ovine FSH-20, National Institute of Diabetes and Digestive and Kidney Disease, Torrance, CA), and 50 ng/μL epidermal growth factor for 23 ± 1 h at 38.5°C in an atmosphere of 5% CO_2_ and air. After maturation, COCs with expanded cumulus cells were fertilized with frozen–thawed, male- or female-sexed sperm, with ≥91% sex-specificity and originating from a single ejaculate (ST Genetics, Navasota, TX) for 18 ± 1 h in F-CDM. The start of sperm-oocyte coincubation was considered time 0 or Day 0. After fertilization, potential zygotes were transferred into holding medium (HCDM-1), vortexed for 90 s to remove cumulus cells, washed, and transferred into equilibrated CDM-1 at 38.5°C in an atmosphere of 5% CO_2_, 5% O_2_, and 90% N_2_. After approximately 56 h of culture in CDM-1, embryos were removed and assessed for cleavage into at least two blastomeres. Cleaved embryos were transferred into equilibrated CDM-2 at the same temperature and gas mix for the remainder of the experiment.

For this study, blastocysts were defined as embryos with a blastocoel >50% of the embryo proper, including embryos considered at the developmental stages of blastocyst, expanded blastocyst, and hatched blastocyst as represented by embryo development stages 6, 7, and 8 by the International Embryo Technology Society [27]. Embryos with a blastocoel <50% of the embryo proper (stage 5) were considered early blastocysts and not included. All blastocysts were removed from CDM-2 on Day 7 after approximately 114 h and considered normal in developmental timing (Normal). The remaining developing embryos, at all developmental stages to early blastocyst, were left in the culture well until Days 8.5–9 or after approximately 144–162 h of culture in CDM2 (Delayed), at which time blastocysts were removed. Initial selection of embryos for further analysis included a real-time assessment of normal morphology and an expansion score of ≥6, indicating the blastocyst to hatched blastocyst stage; therefore, poor-quality and poorly expanded blastocysts were excluded [27]. Blastocysts resulting from oocytes fertilized with sexed sperm with a high probability of producing genetically female embryos (Female) or male embryos (Male) were maintained separately. Embryos from three replicates, including all groups (Normal or Delayed and Male or Female), were used for metabolic and proteomic analyses; two additional replicates were performed to obtain additional blastocysts for metabolic assays. Images of individual embryos were obtained after culture and used for diameter measurements using CellSens software (Olympus, Tokyo, Japan). Using the images, blinded assessments of embryo quality scores (1, excellent to 4, degenerate) were performed and used for statistical analyses [27]. Embryos for metabolic assays were randomly selected from each group and maintained in holding medium (H-CDM) at 4°C for 1.5–2 h prior to metabolic sensor analyses. Embryos for proteomic analyses were obtained from three replicates, with each replicate providing between 5 and 10 pooled embryos per group, totaling 18–24 embryos for each of the four groups (Normal Male, Normal Female, Delayed Male, and Delayed Female). Pooled embryos were washed and snap-frozen in phosphate-buffered saline until proteomic analysis.

### Microsensor analyses oxygen consumption rate, extracellular acidification rate, and reactive oxygen species

An electrochemical multisensor platform was used to assess the metabolic activity of individual blastocysts, including the OCR (indicator of aerobic metabolism), ECAR (indicator of anaerobic metabolism), and hydrogen peroxide production (HPR, indicator of ROS production). The platform consisted of multiple gold working and counter electrodes, along with multiple silver/silver chloride pseudo-reference electrodes for a set of three-electrode electrochemical cells on a glass substrate. The oxygen sensor surface was functionalized with Nafion; the hydrogen peroxide sensor employed a bare gold electrode; and the indium tin oxide pH electrode was micropatterned using photolithography. Additional details regarding sensor fabrication and calibration have been described in previous studies [28, 29].

Blastocysts were warmed to 38.5°C for 15 min prior to analysis. Embryos were transferred to a warmed 3-morpholinopropane-1-sulfonic acid (MOPS)-buffered holding medium (G-MOPS; Vitrolife, Sweden) containing 0.04% fatty acid–free BSA; six blastocysts were individually evaluated in parallel for up to 1 h within a sealed 250 μL microchamber maintained at 38.5°C. Multiple metabolic rates were simultaneously monitored, including the OCR, HPR, and ECAR. Amperometry was used for OCR and HPR measurements, while potentiometry was employed for ECAR assessment. Baselines for each rate were established prior to sample injection into the platform, and the platform started recording the basal rates from each blastocyst 10 min after the injection, allowing minimalization of disturbance from the injection process. Sensor readings were considered complete as soon as the platform detected stable metabolic rates from all six blastocysts; a stable measurement was defined as a maximum deviation of less than 5% over a 3-min window for each metabolic rate measurement.

### Proteomics sample preparation and extraction

Preparation of proteomics samples from pooled embryos was performed at the University of Colorado School of Medicine Proteomics Core in Aurora, CO. Samples were reduced, alkylated, and digested using S-Trap micro filters (Protifi, Huntington, NY) according to the manufacturer’s protocol. Digested peptides were cleaned using Pierce C18 Spin Tips (Thermo Scientific) according to the manufacturer’s protocol, dried in a vacuum centrifuge, and resuspended in 0.1% formic acid (FA) in mass spectrometry–grade water. Peptides were loaded into autosampler vials and analyzed directly using a NanoElute liquid chromatography system (Bruker, Germany) coupled with a timsTOF SCP mass spectrometer (Bruker, Germany). Peptides were separated on a 75 μm i.d. × 15 cm separation column packed with 1.9 μm C18 beads (Bruker, Germany) over a 90-min elution gradient. Buffer A was 0.1% FA in water and buffer B was 0.1% FA in acetonitrile. Instrument control and data acquisition were performed using Compass Hystar (version 6.0) with the timsTOF SCP operating in parallel accumulation–serial fragmentation (PASEF) mode under the following settings: mass range 100–1700 *m/z*, 1/k/0 Start 0.7 V s cm^−2^ End 1.3 V s cm^−2^; ramp accumulation times were 166 ms; capillary voltage was 4500 V, dry gas 8.0 L min^−1^, and dry temp 200°C. The PASEF settings were: 5 MS/MS scans (total cycle time, 1.03 s); charge range 0–5; active exclusion for 0.2 min; scheduling target intensity 20 000; intensity threshold 500; and collision-induced dissociation energy 10 eV.

Fragmentation spectra were searched against the UniProt bovine proteome database using the MSFragger-based FragPipe computational platform [30]. Contaminants and reverse decoys were added to the database automatically*.* The precursor-ion mass tolerance and fragment-ion mass tolerance were set to 15 and 20 ppm, respectively. Fixed modifications were set as carbamidomethyl (C), and variable modifications were set as oxidation (M), two missed tryptic cleavages were allowed, and the protein-level false discovery rate was ≤1%.

### Statistical analysis

For microsensor metabolic measurements, the general linear model was used to compare the effects of sex (Male and Female), developmental timing status (Normal and Delayed), and replicates as well as the interactions among the variables. Tukey adjusted pairwise comparison was used to determine differences among groups (Male Normal, Female Normal, Male Delayed, and Female Delayed). For proteomic analysis, MetaboAnalyst was used to compare protein abundance between groups. Peak intensities were uploaded to MetaboAnalyst, normalized by median, log-transformed (base 10), and autoscaled. Analyses included an unpaired *t*-test to compare the effects of sex or developmental timing status, one-way analysis of variance (ANOVA) to compare all groups, and the Fisher least significant difference (LSD) post hoc test to compare group means. Differences in electron transport complex proteins between Normal and Delayed were evaluated by adding peak intensities in each sample and comparing them with an unpaired *t*-test. Significance was defined as *P* < 0.05, and tendencies were reported for *P* ≤ 0.1.

## Results

### Oxygen consumption, extracellular acidification, and reactive oxygen species production

**Figure 1:**
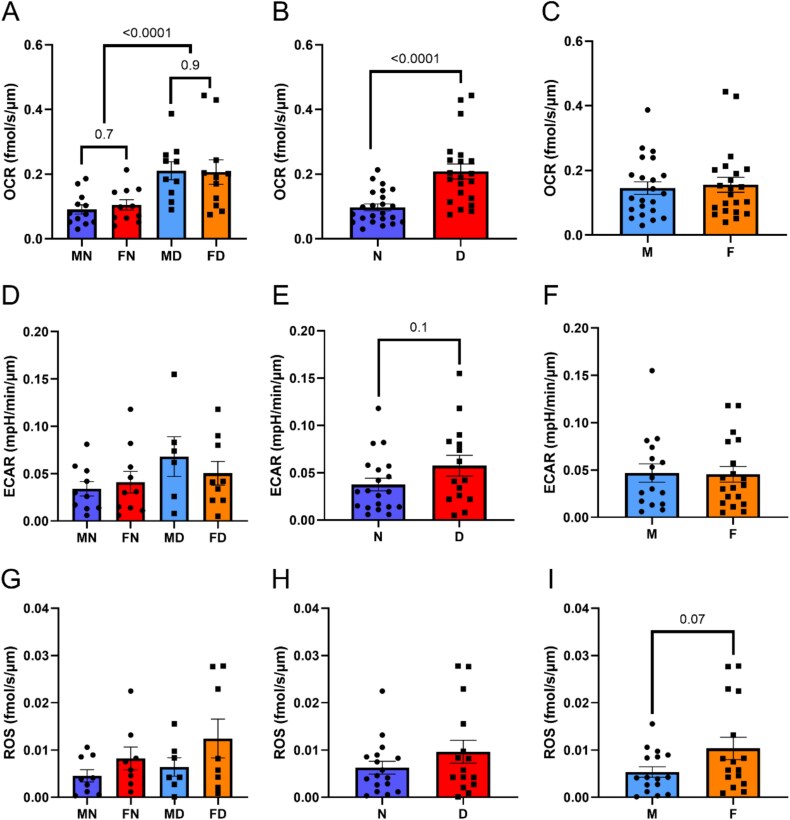
Embryo metabolic assays. Rates of (A–C) oxygen consumption (OCR), (D–F) extracellular acidification (ECAR), and (G–I) hydrogen peroxide production indicative of reactive oxygen species (ROS) in blastocysts forming on day 7 (normal developmental timing, Normal, N, *n* = 24) or days 8.5–9 (delayed development timing, Delayed, D, *n* = 22) and produced using sperm sexed as female (F) or male (M). Five replicates with total embryos for MN (*n* = 13), FN (*n* = 11), MD (*n* = 10), and FD (*n* = 12). Data were compared using the generalized linear model and Tukey-adjusted pairwise comparisons (A, D, G) or unpaired *t*-test (B–C, E–F, H–I). *P*-values ≤0.1 are presented above bars.

Differences in metabolism associated with embryo sex and developmental timing were assessed using an electrochemical, multichannel microsensor. Metabolic readings were obtained for 46 embryos grouped as Female Normal (*n* = 11), Male Normal (*n* = 13), Female Delayed (*n* = 12), or Male Delayed (*n* = 10). Embryo quality grade and diameter, respectively, did not differ by pooled sex (Male, 1.6 ± 0.2 and 184.6 ± 6.3 μm and Female, 2.3 ± 0.1 and 182.1 ± 5.9 μm, *P* > 0.1) or pooled developmental timing (Normal, 1.9 ± 0.1 and 189.1 ± 4.9 μm and Delayed, 2.3 ± 0.2 and 177.1 ± 7.0 μm, *P* > 0.1). There was no effect of replicate on metabolic rates (*P* > 0.2) and no interaction of replicate with sex or developmental timing (*P* > 0.2). However, OCR was affected (*P* < 0.0001) by developmental timing, and sex tended (*P* < 0.07) to influence ROS. No significant interactions were observed for metabolic parameters between sex and developmental timing. For OCR, Delayed Female and Male (*P* = 0.9) and Normal Female and Male (*P* = 0.7) did not differ (Figure 1A). When Male and Female were pooled, the OCR was greater (*P* < 0.0001) in Delayed when compared to Normal (Figure 1B). However, the OCR was similar (*P* = 0.8) between Male and Female (Figure 1C). The ECAR was similar (*P* = 0.4) among all groups, regardless of developmental timing or sex (Figure 1D). When Male and Female were pooled, ECAR tended (*P* = 0.1) to be increased in Delayed when compared to Normal (Figure 1E). The hydrogen peroxide production rate (ROS) was similar among all groups (*P* = 0.3) with no difference for developmental timing. However, ROS tended (*P* = 0.07) to be higher in pooled Female than Male (Figure 1G–I). Although metabolic parameters were not different for Female and Male, embryos capable of forming a blastocyst, but delayed in developmental timing, had a metabolic profile suggestive of either compensatory efforts to increase energy production or a dysregulation of mitochondrial activity.

### Protein differences associated with timing of embryo development

**Figure 2:**
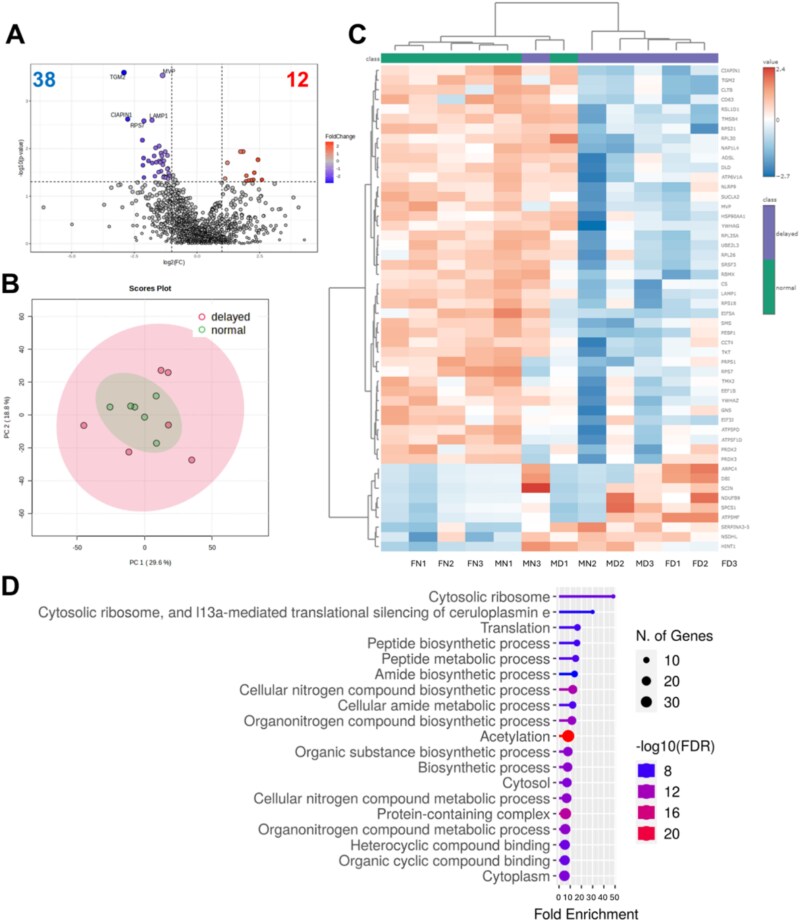
Protein abundance profile of pooled Male and Female embryos considered normal (Normal) or delayed (Delayed) in timing to reach the blastocyst stage of development. (A) Volcano plot indicating fold differences in Delayed relative to Normal. (B) Principal component analysis (PCA) plot with Normal (green) and Delayed (pink). (C) Heatmap and hierarchical clustering of top 50 proteins with significant fold differences for Normal (green bars) and Delayed (purple bars). (D) Enrichment analysis demonstrating similar pathway activity associated with protein differences. Three replicates with a total of 44 Normal and 38 Delayed blastocysts. Data were median-normalized, log2-transformed, auto-scaled, and compared using an unpaired *t*-test with *P* < 0.05. Horizontal line in the volcano plot (A) at *P* < 0.05.

To further explore differences in metabolism associated with normal or delayed embryo developmental timing, Male (*n* = 40 embryos) and Female (*n* = 42 embryos) from three replicates were pooled to compare proteins associated with metabolism in Normal (*n* = 44) and Delayed (*n* = 38). A total of 50 proteins were differentially abundant (*P* < 0.05) between Normal and Delayed (Figure 2, Supplemental Table S1). Of those, 12 were greater and 38 were less abundant in Delayed than Normal (Figure 2A), with more component variability noted in Delayed versus Normal (Figure 2B). Pathway enrichment analysis revealed important functional clusters, with most affected in Delayed and including ribosomal activity, protein metabolism, biosynthetic processes, translation, and acetylation (Figure 2D). Proteins involved with biosynthesis were primarily less abundant in Delayed (*P* < 0.05), including those associated with protein synthesis (ribosomal proteins RPS18, RPL26, RPL35A, RPS7, RPS21, RPL30; eukaryotic translation initiation factor 3 subunit J [EIF3J]; elongation factor 1-beta [EEF1B2]; eukaryotic translation initiation factor 5A-1 [EIF5A]), nucleotide production (adenylosuccinate lyase [ADSL], phosphoribosylpyrophosphate synthetase [PRPS1], transketolase [TKT]), lipogenesis (spermine synthase [SMS]), and ATP production (ATP synthase subunit D [ATP5PD]; ATP synthase F1 subunit delta [ATP5F1D]) (Table 1). Only two anabolic proteins were greater (*P* < 0.02) in Delayed, involving biosynthesis of cholesterol (NAD(P)-dependent steroid dehydrogenase-like [NSDHL]) and ATP (ATP synthase membrane subunit f [ATP5MF]) (Table 1). In addition, two isoforms of peroxiredoxins (PRDX2 and PRDX3), antioxidant enzymes that reduce intracellular peroxides, were less abundant (*P* < 0.04) in Delayed (Table 1). The pro-apoptotic protein, scinderin (SCIN), was greater (*P* = 0.02) in Delayed (Table 1). Proteins involved in the tricarboxylic acid cycle (TCA) cycle, such as citrate synthase (CS), were less abundant (*P* = 0.04) in Delayed, while NADH dehydrogenase 1 beta subcomplex subunit 9 [NDUFB9], a component of complex I of the electron transport system, was greater in Delayed (*P* = 0.03) (Table 1). Overall, protein fold differences were observed in embryos considered normal or delayed in developmental timing, which were consistent with reduced biosynthesis and more metabolic variability in Delayed.

**Table 1:** Pathway association, protein identification, fold difference (FD), and *P*-values associated with differentially abundant proteins for pooled Male and Female embryos considered normal (Normal) or delayed (Delayed) in timing to reach the blastocyst stage of development. Three replicates with total embryos for Normal (*n* = 44) and Delayed (*n* = 38). Fold difference (FD) indicates Delayed relative to Normal.

Pathway	ID	Name	FD	*P*-value
Protein synthesis	RPS18	Ribosomal protein S18	0.44	0.03
	RPL35A	Ribosomal protein L35a	0.46	0.03
	RPL26	Ribosomal protein L26	0.45	0.03
	RPS7	Ribosomal protein S7	0.23	0.003
	RPS21	Ribosomal protein S21	0.31	0.01
	RPL30	Ribosomal protein L30	0.29	0.02
	EIF3J	Eukaryotic translation initiation factor 3 subunit J	0.32	0.02
	EEF1B	Elongation factor 1-beta	0.38	0.01
	EIF5A	Eukaryotic translation initiation factor 5A-1	0.31	0.04
Nucleotide production	ADSL	Adenylosuccinate lyase	0.45	0.04
	PRPS1	Phosphoribosylpyrophosphate synthetase 1	0.22	0.007
	TKT	Transketolase	0.35	0.02
Lipogenesis	SMS	Spermine synthase	0.35	0.03
TCA cycle/ATP production	ATP5PD	ATP synthase subunit D	0.37	0.03
	ATP5F1D	ATP synthase F1 subunit delta	0.26	0.02
	ATP5MF	ATP synthase membrane subunit f	3.33	0.01
	CS	Citrate synthase	0.47	0.04
	NDUFB9	NADH dehydrogenase 1 beta subcomplex subunit 9	4.88	0.03
Stress response	PRDX2	Peroxiredoxin 2	0.40	0.04
	PRDX3	Peroxiredoxin 3	0.22	0.02
Apoptosis	SCIN	Scinderin	2.44	0.02
Cholesterol synthesis	NSDHL	NAD(P)-dependent steroid dehydrogenase–like	1.84	0.01

### Electron transport system complex organization associated with developmental timing

To evaluate potential dysregulation of the electron transport system, as suggested by a greater OCR in Delayed, sums of normalized protein intensities unique to individual electron transport system complexes were compared between Normal and Delayed. Complex II (succinate dehydrogenase) proteins were greater (*P* = 0.02) in Delayed when compared to Normal (Supplemental Figure S2B). Other complexes, including proteins for complex I (Type I NADH dehydrogenase), complex III (CoQH2-cytochrome c reductase), and complex IV (cytochrome c oxidase), tended to be greater (*P* ≤ 0.8) in Delayed when compared to Normal (Supplemental Figure S2A, C, and D). The sum of complex 5 (ATP synthase) proteins, all complexes, and total mitochondrial proteins did not differ (*P* > 0.1) by timing of blastocyst development (Supplemental Figure S2E–G).

### Protein differences associated with embryo sex

To evaluate overall differences in proteomes of Male and Female (*n* = 40 and 42 total embryos, respectively), blastocysts considered normal or delayed in developmental timing were pooled by presumed sex. A total of 45 proteins were greater (*P* < 0.05) in Female when compared to Male, including for pathways such as glucose metabolism (glucose transporter 1 [SLC2A1]; 6-phosphofructokinase [PFKL]); stress response (superoxide dismutase 2 [SOD2]; peroxiredoxin 4 [PRDX4]), mitochondrial function (isocitrate dehydrogenase [NAD] subunit beta [IDH3B]), and biosynthesis (D-dopachrome tautomerase [DDT]; vanin 1 [VNN1]) (Supplemental Table S2). In comparison to Female, Male had greater (*P* < 0.05) abundance of 19 proteins, involving pathways such as apoptosis (14-3-3 protein epsilon [YWHAE]) and biosynthesis (ribosomal protein S3A [RPS3A]; pyrroline-5-carboxylate reductase 3 [PYCR3]; HMG-CoA reductase [HMGCR]; ferrochelatase [FECH]) (Supplemental Table S2 and Supplemental Figure S3). Therefore, differences in protein abundance were observed between Male and Female, although sex differences were not observed in microsensor metabolic analyses.

**Figure 3:**
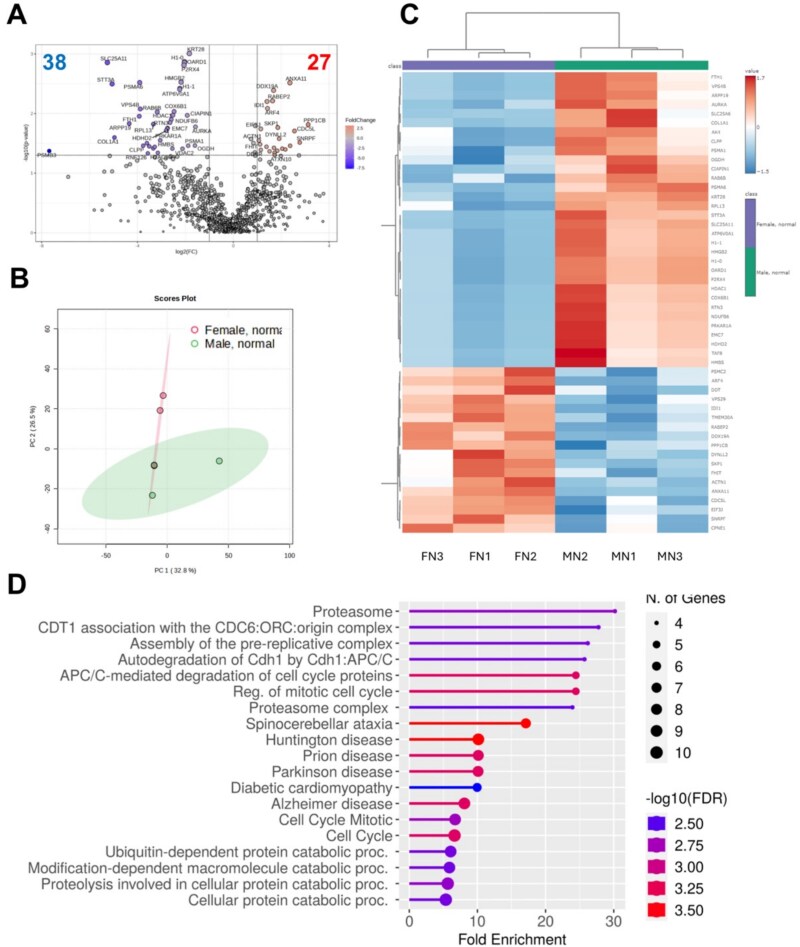
Protein abundance profile of blastocysts with normal timing of development for Male (MN) and Female (FN). (A) Volcano plot indicating fold differences in Female relative to Male. (B) Principal component analysis (PCA) plot with MN (green) and FN (pink). (C) Heatmap and hierarchical clustering of top 50 proteins with significant fold differences. (D) Enrichment analysis demonstrating similar pathway activity associated with protein differences. Three replicates with total embryos for MN (*n* = 20) and FN (*n* = 24). Data were median-normalized, log2-transformed, auto-scaled, and compared using an unpaired *t*-test with *P* < 0.05. Horizontal line in volcano plot (A) at *P* < 0.05.

To assess sex differences associated with blastocysts that developed at a normal (optimal) timing, the proteome of Male or Female blastocysts considered Normal were compared. Between samples from the two groups obtained from three replicates, the abundance of 27 proteins was greater in Female Normal (*n* = 24 total embryos), and 38 proteins were greater in Male Normal (*n* = 20 total embryos, *P* < 0.05, Figure 3). Pathway enrichment analysis of embryos with normal developmental timing for Male and Female revealed possible differences in proteins that are involved with proteolysis (*n* = 10) and cell cycle regulation (*n* = 10); however, very few metabolic proteins were differentially abundant between Male and Female embryos with normal timing of development to the blastocyst stage (Supplemental Table S3). Protein abundance of cytochrome c oxidase subunit 6B1 (COX6B1), a component of complex IV in the electron transport system, and mitochondrial 2-oxoglutarate/malate carrier protein (SLC25A11) were both greater in embryos with normal developmental timing for Male when compared to Female (*P* < 0.05, Supplemental Table S3). Although differences were observed in the proteome of Male and Female embryos with normal developmental timing, protein differences specifically associated with metabolism were minimal and supported microsensor metabolic findings.

### Sex-specific differences associated with developmental timing

**Figure 4:**
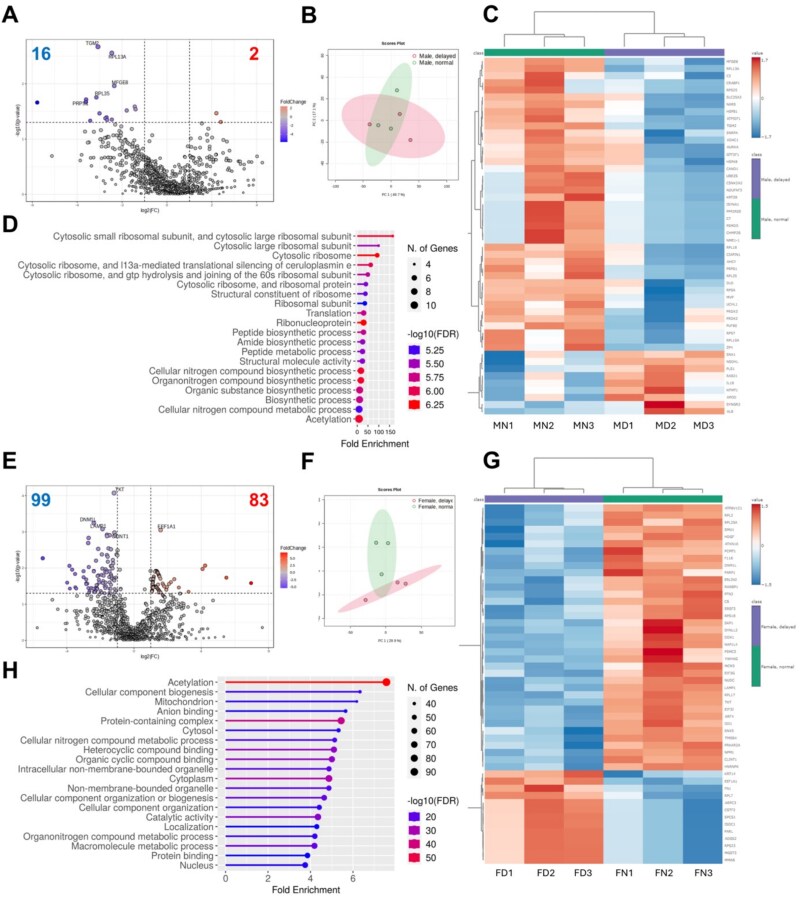
Protein abundance profile of (A–D) Male embryos with normal (MN) and delayed (MD) timing of blastocyst development and (E–H) Female embryos with normal (FN) and delayed (FD) timing of blastocyst development. (A, E) Volcano plot indicating fold differences in Delayed relative to Normal, (B, F) Principal component analysis (PCA) plot with (B) MN (green) and MD (pink) or (F) FN (green) and FD (pink), (C, G) Heatmap and hierarchical clustering of top 50 proteins with significant fold differences for Normal (green bars) or Delayed (purple bars), (D, H) Enrichment analysis demonstrating similar pathway activity associated with protein differences. Three replicates with total embryos for MN (*n* = 20), MD (*n* = 20), FN (*n* = 24), and FD (*n* = 18). Data were median-normalized, log2-transformed, auto-scaled, and compared using an unpaired *t*-test with *P* < 0.05. Horizontal line in the volcano plot (A) at *P* < 0.05.

Proteomes of blastocysts considered to be Normal or Delayed from Male and Female were compared to determine if sex-specific differences were associated with developmental timing to the blastocyst stage. For Male Delayed relative to Male Normal, respectively, 16 and 2 proteins had greater or less protein abundance (*P* < 0.05, Figure 4, Supplemental Table S4) primarily involving protein and nucleotide biosynthesis and including less phosphoribosylpyrophosphate synthetase 1 (PRPS1) and ribosomal proteins (RPS7, RPL35, RPL13A, RPL18, RPL10A) (*P* < 0.05, Table 2). In contrast, substantially more differences were observed for Female, with 99 less and 83 greater protein abundance for Female Delayed when compared to Female Normal (*P* < 0.05, Figure 4, Supplemental Table S5). Pathway enrichment analysis of Normal and Delayed for Female revealed differences in pathways involved with mitochondrial function (*n* = 39), cellular component biogenesis (*n* = 40), and various macromolecule metabolic processes (*n* = 63) (Figure 4H). In Female, Delayed when compared to Normal had greater abundance of stress response proteins including glutathione S-transferase 3 (MGST3), phospholipid hydroperoxide glutathione peroxidase (GPX4), and peroxiredoxin-4 (PRDX4) (*P* < 0.05, Table 2). Proteins involved in protein synthesis also differed for Female Delayed versus Normal, with greater (elongation factor 1-alpha 1 [EEF1A1]; ribosomal proteins RPL7, RPS23, RPS25, RPL24) and less (ribosomal proteins RPL17, RPL3, RPL35A; eukaryotic translation initiation factor 3 subunit J [EIF3J], eukaryotic translation initiation factor 2 subunit 1 [EIF2S2]) proteins (*P* < 0.05, Table 2). For Female with normal versus delayed blastocyst developmental timing, abundance of enzymes involved with lipid metabolism was both greater (very-long-chain enoyl-CoA reductase [TECR]; ethanolamine-phosphate cytidylyltransferase [PCYT2]; diphosphomevalonate decarboxylase [MVD]) and less (isopentenyl-diphosphate delta-isomerase 1 [IDI1]; geranylgeranyl pyrophosphate synthase [GGPS1]) (*P* < 0.05, Table 2). Similar to the trends observed with combined Male and Female, the abundance of proteins involved in the electron transport system were generally greater in Female Delayed when compared to Normal, including ATP synthase subunit f (ATPMF), cytochrome b-c1 complex subunit 9 (UQCR10), NADH dehydrogenase [ubiquinone] 1 alpha subcomplex assembly factor 4 (NDUFAF4), NADH–ubiquinone oxidoreductase chain 5 (MT-ND5), and cytochrome c oxidase subunit 7A-related protein, mitochondrial (COX7A2L), although cytochrome b-c1 complex subunit 7 (UQCRB) was greater in Female Normal versus Delayed (*P* < 0.05, Table 2). In addition, CS and succinate-CoA ligase (SUCLA2) were greater (*P* ≤ 0.02) in Female Normal when compared to Female Delayed (Table 2). Sex-associated differences in the proteomes of Normal and Delayed were observed, with substantially more variation noted for Female than Male.

**Table 2:** Pathway association, protein identification, fold difference (FD), and *P*-values associated with differentially abundant proteins for pooled blastocysts considered Normal and Delayed in developmental timing for Male or Female. Three replicates with total embryos for Male Normal (*n* = 20), Male Delayed (*n* = 20), Female Normal (*n* = 24), and Female Delayed (*n* = 18).

Pathway	ID	Name	FD	*P*-value
Male Delayed relative to Male Normal				
Protein synthesis	RPL13A	Large ribosomal subunit protein uL13	0.18	0.003
	RPL35	Large ribosomal subunit protein uL29	0.11	0.02
	RPL10A	Large ribosomal subunit protein uL16z	0.15	0.04
	RPL18	Large ribosomal subunit protein eL18	0.14	0.04
	RPS7	Small ribosomal subunit protein eS7	0.16	0.05
Nucleotide production	PRPS1	Ribose-phosphate pyrophosphokinase 1	0.08	0.02
Female Delayed relative to Female Normal				
Protein synthesis	EEF1A1	Elongation factor 1-alpha 1	3.00	0.0009
	RPL17	Large ribosomal subunit protein uL22	0.46	0.001
	EIF3J	Eukaryotic translation initiation factor 3 subunit J	0.15	0.001
	RPL3	Large ribosomal subunit protein uL3	0.27	0.004
	RPS18	Small ribosomal subunit protein uS13	0.34	0.007
	RPL7	Large ribosomal subunit protein uL30	16.88	0.01
	RPL35A	RPL35A ribosomal protein L35a	0.19	0.01
	RPS23	Small ribosomal subunit protein uS12	2.52	0.01
	RPS25	Small ribosomal subunit protein eS25	2.26	0.02
	RPL24	Large ribosomal subunit protein eL24	45.10	0.02
	EIF2S2	Eukaryotic translation initiation factor 2 subunit 1	0.26	0.05
TCA cycle/ ATP production	CS	Citrate synthase	0.51	0.003
	SUCLA2	Succinate–CoA ligase	0.26	0.02
	ATP5MF	ATP synthase subunit f, mitochondrial	2.21	0.02
	UQCR10	Cytochrome b-c1 complex subunit 9	2.12	0.03
	NDUFAF4	NADH dehydrogenase [ubiquinone] 1 alpha subcomplex assembly factor 4	2.22	0.03
	MT-ND5	NADH–ubiquinone oxidoreductase chain 5	2.53	0.03
	UQCRB	Cytochrome b-c1 complex subunit 7	0.31	0.04
	COX7A2L	Cytochrome c oxidase subunit 7A-related protein, mitochondrial	2.69	0.04
Lipid metabolism	IDI1	Isopentenyl-diphosphate delta-isomerase 1	0.15	0.002
	GGPS1	Geranylgeranyl pyrophosphate synthase	0.13	0.02
	TECR	Very-long-chain enoyl-CoA reductase	2.17	0.02
	PCYT2	Ethanolamine-phosphate cytidylyltransferase	2.40	0.03
	MVD	Diphosphomevalonate decarboxylase	2.20	0.03
Stress response	MGST3	Glutathione S-transferase 3, mitochondrial	2.54	0.01
	HSP90AA	Heat shock protein HSP 90-alpha 1	0.39	0.02
	GPX4	Phospholipid hydroperoxide glutathione peroxidase	2.77	0.02
	PRDX4	Peroxiredoxin-4	5.26	0.02

**Figure 5:**
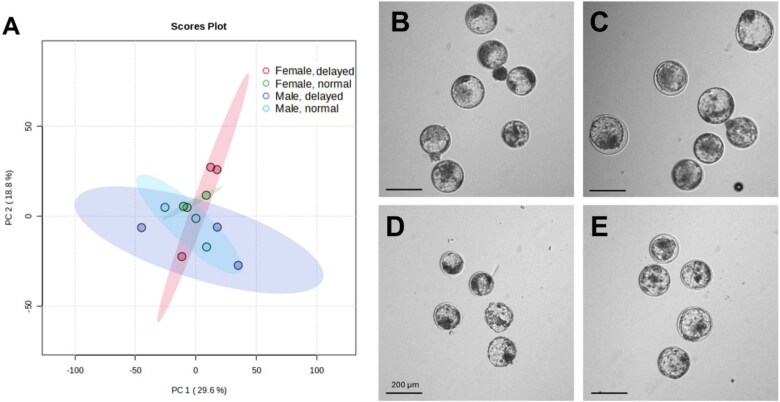
Effect of sex and developmental status on the protein abundance profile of pooled blastocysts from Female Normal (FN), Female Delayed (FD), Male Normal (MN), and Male Delayed (MD). (A) Principal component analysis (PCA) plot with FN (green), FD (pink), MN (blue), and MD (purple). Representative images of groups of blastocysts in (B) Male Normal, (C) Female Normal, (D) Male Delayed, and (E) Female Delayed. Scale bar at 200 μm.

To better isolate possible sex differences associated with delayed embryo development, all groups (Normal, Delayed, Male, and Female) were compared by ANOVA (Figure 5, Supplemental Table S6, Supplemental Figure S4). In Female, delayed embryo development timing was uniquely associated with greater (*P* < 0.05) abundance of proteins involved with the stress response (peroxiredoxin-4 [PRDX4]; superoxide dismutase 2 [SOD2]; glutathione S-transferase 3 [MGST3]), anion transport (voltage-dependent anion-selective channel VDAC2, VDAC3), and protein biosynthesis (ribosomal protein S30 [FAU]; ribosomal protein S25 [RPS25]; eukaryotic translation elongation factor 1 alpha 1 [EEF1A1]) (Supplemental Table S6). When compared to Female Delayed, Female Normal had a greater (*P* = 0.04) abundance of carnitine palmitoyltransferase II (CPT2), an enzyme important for fatty acid metabolism (Supplemental Table S6). Male Delayed had less abundant proteins involved in nucleotide synthesis (phosphoribosyl pyrophosphate synthetase 1 [PRPS1]), protein biosynthesis (ribosomal proteins RPS7, RPSA), and anion transport (Voltage-dependent anion-selective channel 1 [VDAC1]) (*P* < 0.05) when compared to Male Normal (Supplemental Table S6).

## Discussion

Intrinsic differences in embryo developmental potential are not yet fully understood. Not all embryos that develop to the blastocyst stage are viable, although faster-developing embryos are typically associated with greater developmental potential [7–9]. In addition, sex of the bovine embryo has been postulated to be associated with speed of development and metabolism [31]. In the present study, the metabolism and proteome of bovine blastocysts that developed during a normal or delayed timeline were compared to determine potential intrinsic causes of delayed preimplantation development, with a focus on imbalances of the catabolic and anabolic processes that are required in the rapidly growing embryo.

The metabolic regulation of cell proliferation is a hallmark of biological processes such as fetal development, cancer, and tissue repair. Metabolic pathways, such as glycolysis, oxidative phosphorylation, and amino acid metabolism, are dynamically modulated to support the energetic and biosynthetic demands of proliferating cells [32]. Dysregulation of these processes can lead to impaired growth, ultimately resulting in reduced viability [10]. Metabolic adaptations such as the Warburg effect have been hypothesized to play a role in embryos [33], as studies of proliferative cell types indicate that redox balance and anabolic metabolism are of greater importance than ATP production during periods of rapid growth [34]. Respirometry and proteomic data from the present study provide evidence that blastocysts that are delayed in development deviate from these metabolic adaptations in comparison to blastocysts developing at a normal time interval, although the groups of blastocysts did not significantly differ in developmental stage, size, or morphology grades. Blastocysts delayed in development had a significantly higher rate of oxygen consumption and a clear trend toward higher protein expression of electron transport system (ETS) complexes than faster-developing embryos, consistent with an upregulation of oxidative metabolism. This may indicate that delayed blastocysts could be exhibiting excessive catabolic activity; however, expression of citrate synthase, which catalyzes entry of acetyl-CoA into the TCA cycle that supplies reducing equivalents to the ETS, was lower in delayed embryos. Although not evaluated in the present study, these seemingly paradoxical findings could reflect a rewiring of TCA cycle flux to support greater generation of reducing equivalents from CS-independent pathways, such as the malate–aspartate shuttle, known to be important for biosynthetic metabolism and redox homeostasis in developing embryos [35]. Additional studies are recommended to explore this possibility and elucidate the developmental implications of increased oxidative metabolism in delayed blastocysts.

The quiet embryo hypothesis suggests that lower metabolic activity during the preimplantation stage is associated with greater implantation potential. This has been demonstrated in several studies, including reduced pyruvate metabolism and amino acid turnover in early embryos as a predictor of viability and euploidy [36–39]. However, the original interpretation of the quiet embryo hypothesis has been modified to consider an upper and lower limit to account for embryos with markedly low metabolic activity, with studies correlating both low and high pyruvate consumption to be detrimental to blastocyst formation [16, 40]. Delayed blastulation is a predictor of reduced implantation, euploidy, and pregnancy outcomes in humans [9, 41]. Metabolic results of the present study provide novel support to the quiet embryo hypothesis, as blastocysts delayed in development had a greater oxygen consumption rate suggestive of more aerobic metabolism and energy production; in contrast, embryos with a “quieter” metabolism appeared to develop at a faster rate. The metabolic findings suggest an imbalance of anabolic and catabolic activity in blastocysts that develop on a delayed timeline.

In the present study, embryo proteomes supported metabolism results. Biosynthetic proteins involved in the production of nucleotides, proteins, and lipids were less abundant in blastocysts delayed versus normal in developmental timing. Protein biosynthesis and amino acid uptake are highly active for the blastocyst when compared to earlier-stage embryos [42]. Proteins that make up ribosomes and are involved in the recruitment of protein and mRNA components to ribosomes [43, 44] were lower in delayed-developing blastocysts, suggesting reduced translation. The rate of protein synthesis is highly correlated with cell proliferation, as protein synthesis is regulated by the cell cycle, although translation can also affect the progression of the cell cycle [45]. It is not clear whether the slow growth, and consequently altered cell cycle, was responsible for the decreased abundance of protein-synthesizing proteins or whether lower protein biosynthetic enzymes induced slow growth. Regardless, the expression of ribosomal RNA and protein-synthesizing genes increase dramatically at the blastocyst stage, and they are critical for the increased rate of ribosome biosynthesis in the later stages of preimplantation development [46, 47]. Therefore, the observed decrease in ribosomal activity could have influenced the rate of growth and contributed to delaying blastocyst formation.

Nucleotide synthesis is closely associated with embryo growth, as cells must replicate DNA and produce various RNAs to successfully divide and differentiate [48, 49]. Phosphoribosepyrophosphate (PRPP), a major component of nucleotide molecules and a product of the pentose phosphate pathway, is derived primarily from carbohydrates, such as glucose and fructose [50]. Transketolase (TKT) is critical for the non-oxidative production of ribulose-5-phosphate, a precursor of PRPP [51]. Slower-developing blastocysts had a lower abundance of PRPS1 and TKT, thus indicating a potential reduction in PRPP synthesis and impaired nucleotide synthesis. Dysfunction of the pentose phosphate pathway in embryos is associated with abnormal cell division and developmental arrest, due to reduced production of nucleotides [52]. However, the pentose phosphate pathway is also critical for redox control through generation of nicotinamide adenine dinucleotide phosphate (NADPH), which is essential for lipid, nucleotide, and amino acid synthesis [32, 53]. In the present study, activity of the pentose phosphate pathway was not directly investigated; however, the finding suggests that further investigation into this area should be considered to more completely understand delayed embryo development.

The complete extent of sex-associated differences in embryo metabolism is not yet known, although individual embryo metabolism has not been thoroughly investigated. In the present study, differences in the OCR and ECAR between male and female blastocysts were not observed; however, proteomic data suggested that delayed development may have unique causes or responses depending on embryo sex. Certain differentially abundant proteins aligned with previous studies of embryo sex and metabolism, such as increased glycolytic activity in female mouse embryos [54, 55], consistent with elevated SLC2A1 and PFKL in Female when compared to Male in the present study. Blastocysts produced using intracytoplasmic sperm injection can be disproportionately male and tend to grow faster, although they have higher rates of aneuploidy in humans [56]. Our study did not find many differentially abundant metabolic enzymes between male and female embryos, especially when comparing embryos with normal developmental timing. This was expected and supports our sensor data indicating no differences in the OCR or ECAR between the sexes. However, delayed development in female embryos seemed more consistently associated with mitochondrial dysfunction and stress, while male delayed embryos seemed to have less biosynthetic proteins and less differences between Normal and Delayed. Two peroxiredoxins were decreased in delayed developing blastocysts, indicating that they could have reduced tolerance to oxidative stress. Interestingly, female delayed blastocysts had a greater abundance of several antioxidant enzymes, although it remains to be determined if female embryos are more capable of handling oxidative stress than male embryos. Although currently impractical, sex-specific embryo culture techniques, especially regarding differential glucose uptake and tolerance to stress, may exist in the future to optimize the metabolic demands of male and female embryos.

In the present study, it is unclear when the onset of metabolic differences occurred between normal and slow-growing embryos. The composition of the culture medium and incubation conditions for in vitro embryo culture can influence growth, epigenetics, and viability [17, 18], as will gamete quality [57]. To minimize these effects in the present study, embryos from IVF replicates were assigned to all metabolic and proteome groups, and sexed semen from the same ejaculate of a single bull was used with a proven high rate of sex specificity. While use of a single bull for the entire study limited variability among endpoints, certain outcomes may have been influenced by genetics or epigenetics. Therefore, additional pathways involved in delayed embryo development could be identified using a larger sample number with multiple bulls. Culture conditions were kept consistent, although embryos considered delayed remained in the same media well (500 μL) for the slightly longer interval. Therefore, the primary causes of delayed blastocyst formation in the present study were probably due to intrinsic embryo differences, potentially associated with gamete quality, fertilization timing, or genetic factors.

In conclusion, the results of the present study demonstrated metabolic and proteomic differences between bovine blastocysts that developed on a normal or delayed timeline. Most notably, the balance of anabolic and catabolic embryo activity differed—with normal-developing blastocysts having a “quieter” metabolism associated with greater biosynthesis and delayed blastocysts having greater catabolism and oxidative stress. These data suggest that delayed blastocyst development is associated with an imbalance in the regulation of catabolic and anabolic metabolism, prioritizing energy production over biosynthetic needs. This can explain why some embryos do not undergo cell proliferation and differentiation as quickly as more viable embryos, and it highlights the importance of biosynthetic processes over catabolic activity in embryo development. Although differences in metabolism-associated proteins with embryo sex were limited in blastocysts developing over a normal timeline, more sex-associated variation was observed in embryos delayed in development, indicating potential differences in causative or compensatory mechanisms. Overall, metabolic activity associated with impaired cell proliferation in mammalian embryos was observed in the present study. Future investigations into the regulation of catabolic and anabolic activity in embryos and the causes of disturbances to these pathways may further identify causes of slow embryo growth and provide a basis for culture systems to improve their viability.

## Supplementary Material

BOR_Supplemental_Information_Delayed_070824_ioaf058

## Data Availability

The data underlying this article are available in the article and its online supplemental material.
