# Supplementary material for: Delayed blastocyst development is associated with altered metabolism and proteome in male and female bovine embryos
Source: Biol Reprod. 2025 Mar 21;112(6):1072–85. doi: 10.1093/biolre/ioaf058 (PMC12191644; doi:10.1093/biolre/ioaf058)
Supplement: BOR_Supplemental_Information_Delayed_070824_ioaf058 [file bor_supplemental_information_delayed_070824_ioaf058.docx]

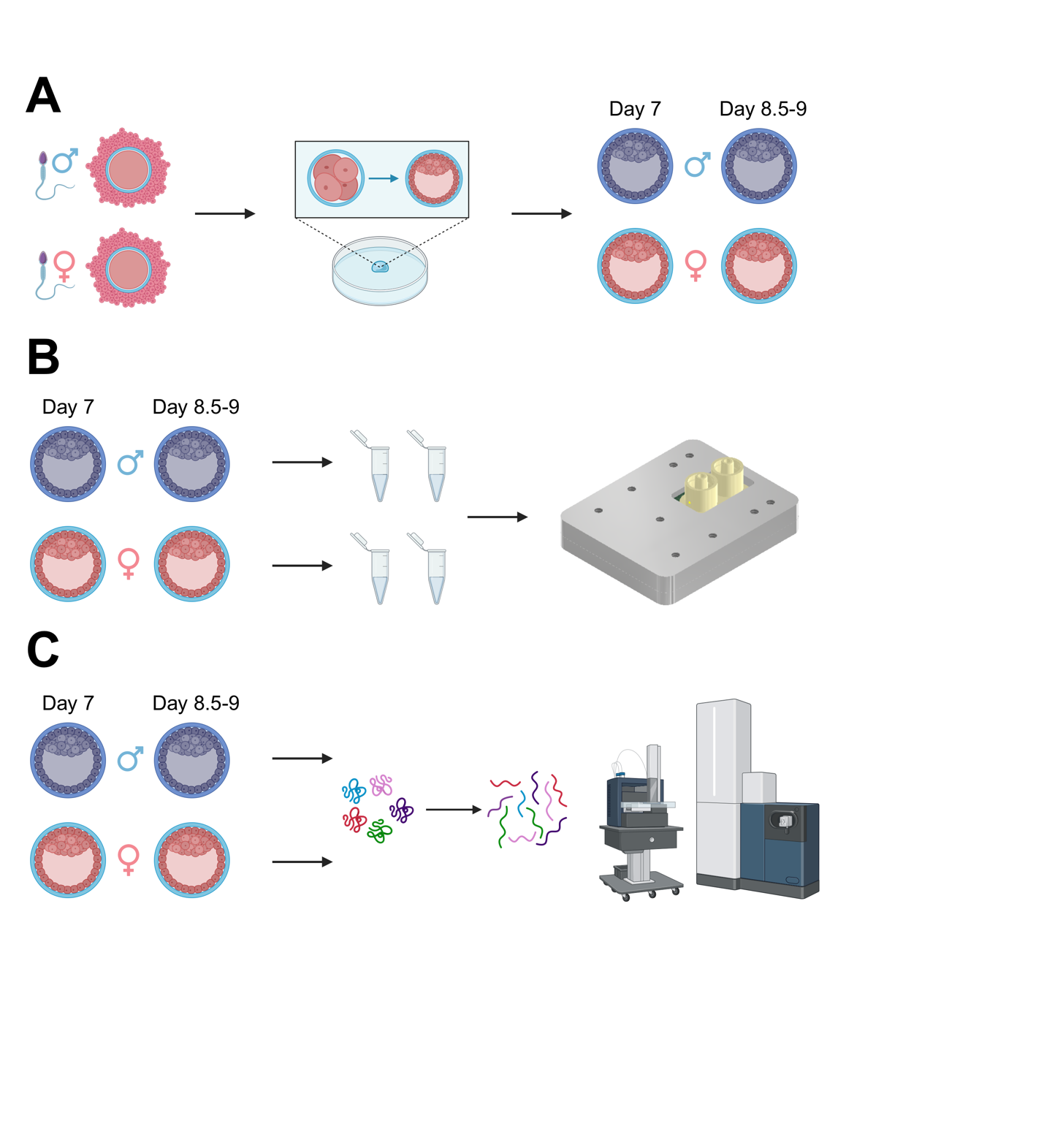


**Supplemental Figure S1**: Experimental design and methodology. (**A**) In vitro matured cumulus oocyte complexes were fertilized with male- or female-sexed sperm. All embryos that reached an expanding blastocyst stage were removed from culture on day 7, with the embryo development timing considered normal (Normal); embryos at an earlier stage of development on Day 7, remained in culture until day 8.5 to 9, with blastocyst development timing considered delayed (Delayed). (**B**) Male and female blastocysts considered Normal or Delayed were placed in a microchamber for the analysis of oxygen consumption rate (OCR), extracellular acidification rate (ECAR), and hydrogen peroxide production rate, indicative of reactive oxygen species production (ROS). (**C**) Additional male and female blastocysts considered Normal or Delayed were pooled for proteomic analysis.


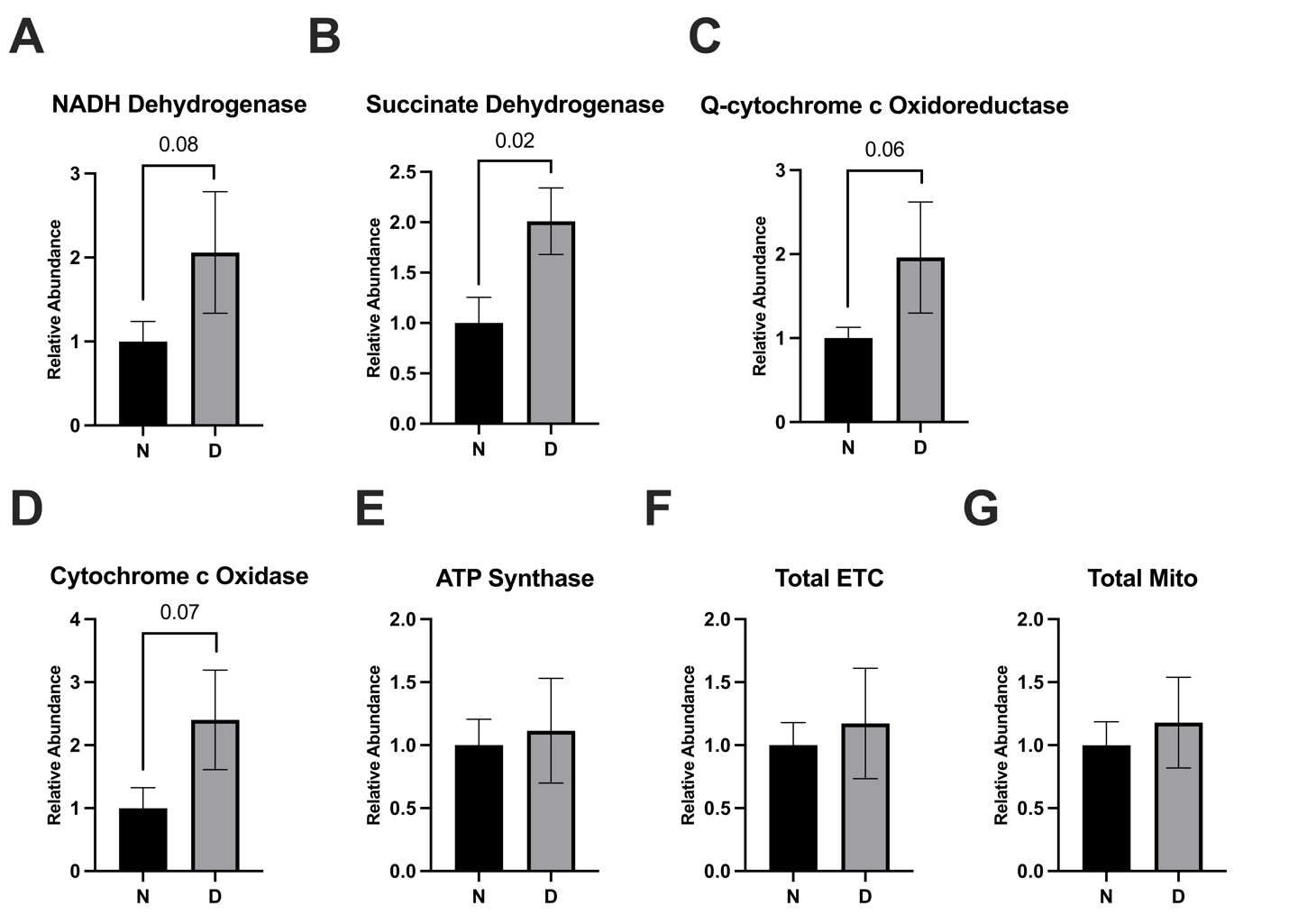


**Supplemental Figure S2:** Means ± SEM for combined normalized intensity of proteins, from embryos considered normal (N) or delayed (D) in developmental timing, composing complexes I-V of the electron transport system: (**A**) Complex 1: NADH dehydrogenase, (**B**) Complex II: succinate dehydrogenase, (**C**) Complex III: Q-cytochrome c oxidoreductase, (**D**) Complex IV: cytochrome c oxidase, (**E**) Complex V: ATP synthase, (**F**) Combined intensity of all complexes, and (**G**) Total mitochondrial protein. Three replicates for a pooled total of embryos for Normal (n=44) and Delayed (n=38). Data were compared using unpaired t-test with p-values above bars for differences p<0.1.


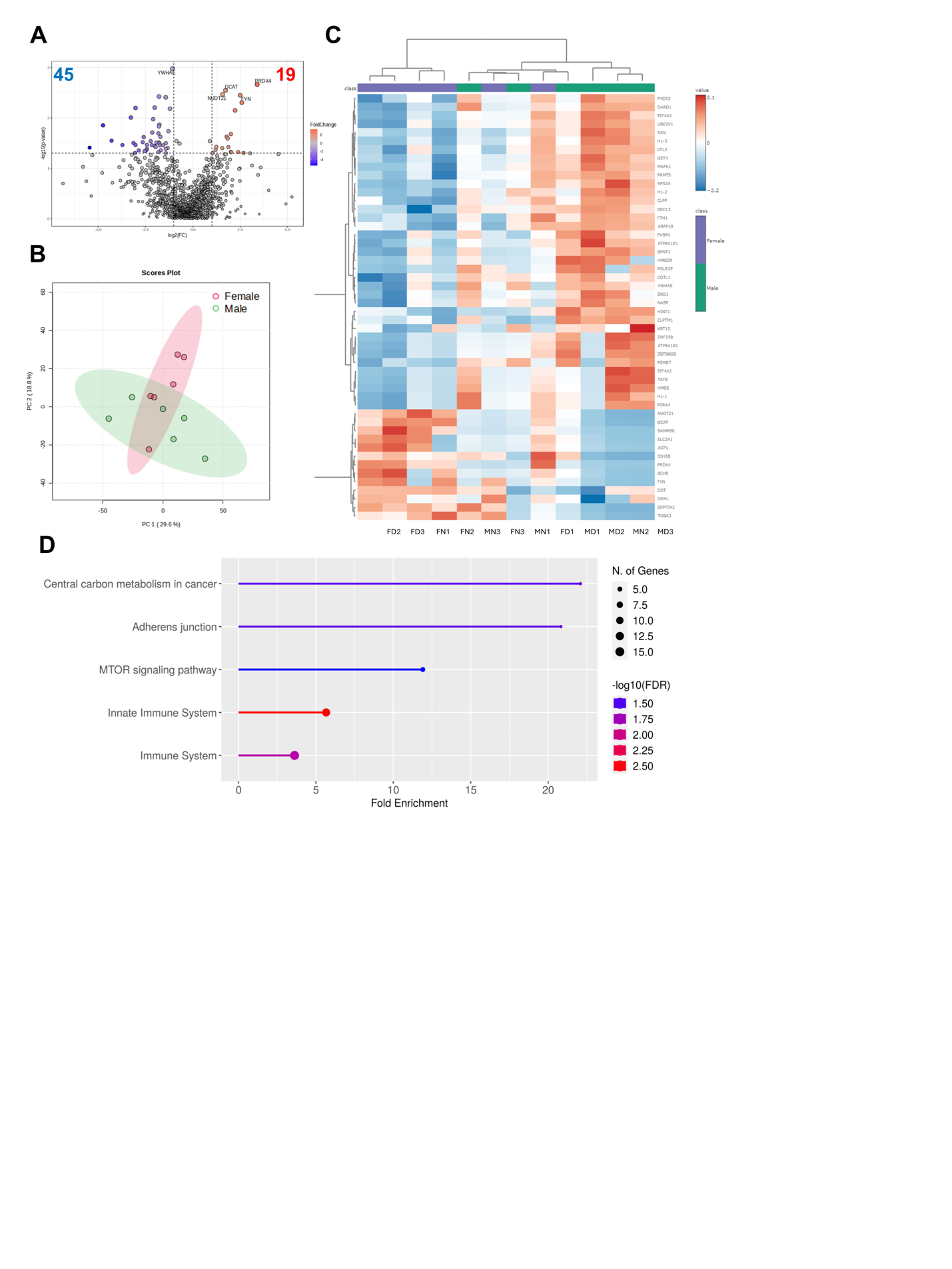


**Supplemental Figure S3.** Protein abundance profile of pooled Male or Female blastocysts with normal and delayed development timing. (**A**) Volcano plot indicating fold differences in Female relative to Male, (**B**) Principal component analysis (PCA) plot with Male (green) and Female (pink), (**C**) Heatmap and hierarchical clustering of 50 proteins with significant fold differences, (**D**) Enrichment analysis demonstrating similar pathway activity associated with protein differences. Three replicates for a total of 40 Male and 42 Female embryos. Data were median normalized, log2 transformed, auto scaled, and compared using unpaired t-test with p<0.05. Horizontal line in volcano plot (**A**) at p<0.05.


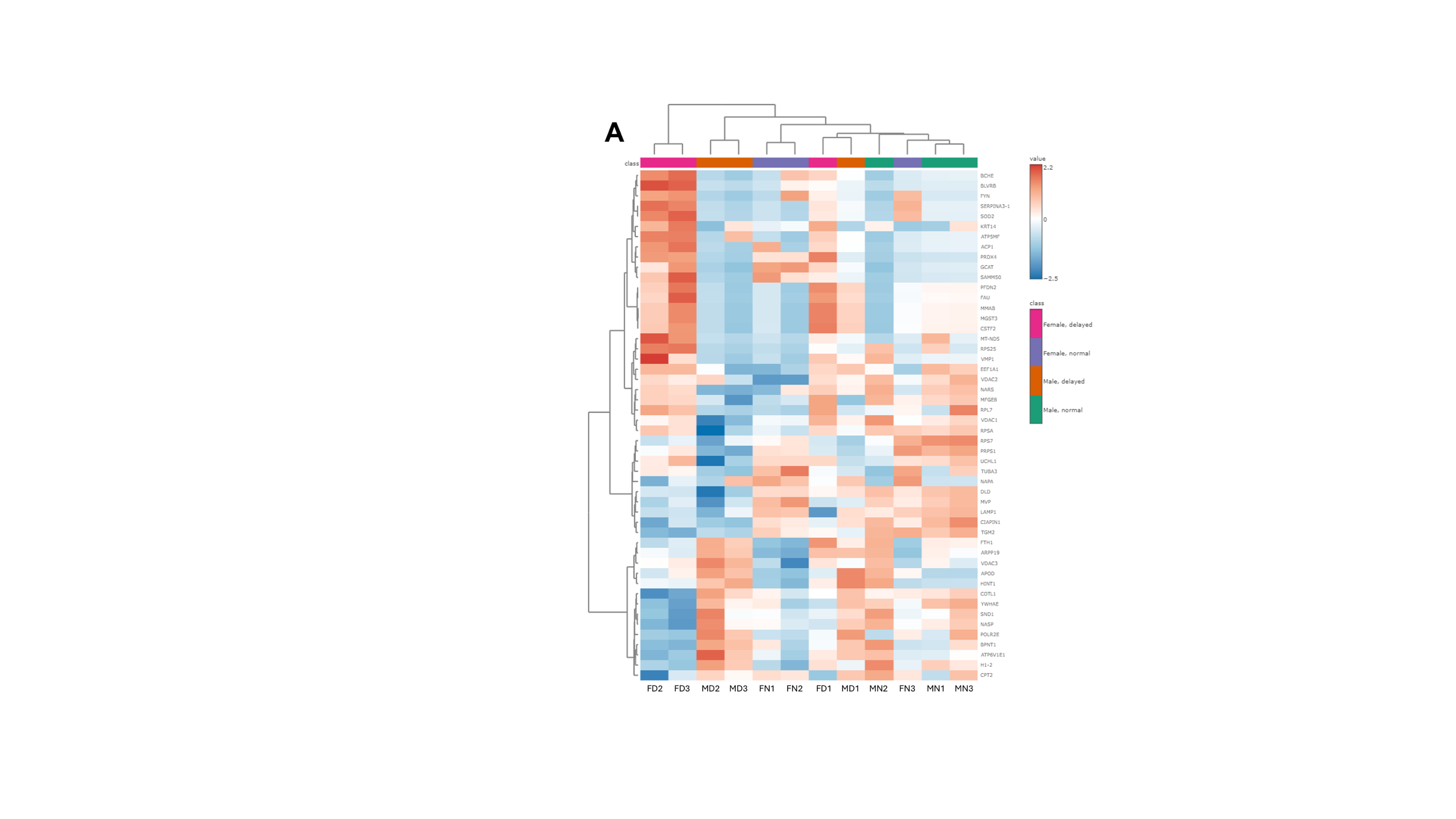


**Supplemental Figure S4.** Effect of sex and developmental status on the protein abundance profile of pooled blastocysts from Female Normal (FN), Female Delayed (FD), Male Normal (MN), and Male Delayed (MD). (**A**) Heatmap and hierarchical clustering of 50 proteins with significant fold differences. Three replicates with total embryos for MN (n=20), FN (n=24), MD (n=20) and FD (n=18). Data was median normalized, log2 transformed, auto scaled, and compared using one-way ANOVA and Fisher’s LSD with p<0.05.

**Supplemental Table S1:** Protein identification, fold difference (FD), and p-values associated with differentially abundant proteins for pooled Male and Female embryos considered normal (Normal) or delayed (Delayed) in timing to reach the expanding blastocyst stage of development. Three replicates with total embryos for Normal (n=44) and Delayed (n=38). Fold difference (FD) indicates Delayed relative to Normal.

| **ID** | **Name** | **FD** | **P-value** |
| --- | --- | --- | --- |
| TGM2 | Protein-glutamine gamma-glutamyltransferase 2 | 0.13 | 0.0003 |
| MVP | Major vault protein | 0.39 | 0.0003 |
| CIAPIN1 | Anamorsin | 0.15 | 0.0024 |
| LAMP1 | Lysosome-associated membrane glycoprotein 1 | 0.29 | 0.0025 |
| RPS7 | Small ribosomal subunit protein eS7 | 0.23 | 0.0026 |
| PRPS1 | Ribose-phosphate pyrophosphokinase 1 | 0.22 | 0.0066 |
| SUCLA2 | Succinate--CoA ligase [ADP-forming] subunit beta, mitochondrial | 0.35 | 0.0089 |
| DLD | Dihydrolipoyl dehydrogenase, mitochondrial | 0.32 | 0.0097 |
| NSDHL | Sterol-4-alpha-carboxylate 3-dehydrogenase, decarboxylating | 3.58 | 0.0116 |
| ATP5MF | ATP synthase subunit f, mitochondrial | 3.33 | 0.0116 |
| TMSB4 | Thymosin beta-4 | 0.42 | 0.0116 |
| NLRP9 | NACHT, LRR and PYD domains-containing protein 9 | 0.37 | 0.0124 |
| PEBP1 | Phosphatidylethanolamine-binding protein 1 | 0.44 | 0.0139 |
| EEF1B | Elongation factor 1-beta | 0.38 | 0.0142 |
| RPS21 | Small ribosomal subunit protein eS21 | 0.31 | 0.0143 |
| TMX2 | Thioredoxin-related transmembrane protein 2 | 0.23 | 0.0158 |
| SCIN | Scinderin | 5.41 | 0.0171 |
| RSL1D1 | Ribosomal L1 domain-containing protein 1 | 0.38 | 0.0178 |
| UBE2L3 | Ubiquitin-conjugating enzyme E2 L3 | 0.40 | 0.0180 |
| ATP5F1D | ATP synthase subunit delta, mitochondrial | 0.26 | 0.0181 |
| TKT | Transketolase | 0.35 | 0.0183 |
| EIF3J | Eukaryotic translation initiation factor 3 subunit J | 0.32 | 0.0193 |
| NAP1L4 | Nucleosome assembly protein 1-like 4 | 0.36 | 0.0194 |
| SPCS1 | Signal peptidase complex subunit 1 | 2.32 | 0.0198 |
| RPL30 | Large ribosomal subunit protein eL30 | 0.29 | 0.0200 |
| SRSF3 | Serine/arginine-rich splicing factor 3 | 0.39 | 0.0218 |
| PRDX3 | Thioredoxin-dependent peroxide reductase, mitochondrial | 0.22 | 0.0224 |
| RPL26 | Large ribosomal subunit protein uL24 | 0.45 | 0.0262 |
| RPS18 | Small ribosomal subunit protein uS13 | 0.44 | 0.0267 |
| ATP5PD | ATP synthase subunit d, mitochondrial | 0.37 | 0.0299 |
| SMS | Spermine synthase | 0.35 | 0.0311 |
| NDUFB9 | NADH dehydrogenase [ubiquinone] 1 beta subcomplex subunit 9 | 4.88 | 0.0321 |
| RPL35A | Large ribosomal subunit protein eL33 | 0.46 | 0.0327 |
| RBMX | RNA-binding motif protein, X chromosome | 0.52 | 0.0363 |
| SERPINA3-5 | Serpin A3-5 | 3.92 | 0.0371 |
| CS | Citrate synthase | 0.47 | 0.0378 |
| PRDX2 | Peroxiredoxin-2 | 0.40 | 0.0380 |
| EIF5A | Eukaryotic translation initiation factor 5A-1 | 0.31 | 0.0381 |
| YWHAZ | 14-3-3 protein zeta/delta | 0.44 | 0.0393 |
| CLTB | Clathrin light chain B | 0.42 | 0.0395 |
| ATP6V1A | V-type proton ATPase catalytic subunit A | 0.41 | 0.0399 |
| YWHAG | 14-3-3 protein gamma | 0.40 | 0.0402 |
| CD63 | CD63 antigen | 0.23 | 0.0405 |
| GNS | N-acetylglucosamine-6-sulfatase | 0.42 | 0.0407 |
| HINT1 | Adenosine 5'-monophosphoramidase HINT1 | 2.18 | 0.0426 |
| ADSL | Adenylosuccinate lyase | 0.45 | 0.0431 |
| CCT4 | T-complex protein 1 subunit delta | 0.51 | 0.0433 |
| DBI | Acyl-CoA-binding protein | 4.76 | 0.0450 |
| ARPC4 | Actin-related protein 2/3 complex subunit 4 | 6.03 | 0.0451 |
| HSP90AA1 | Heat shock protein HSP 90-alpha | 0.55 | 0.0455 |
| ARPC3 | Actin-related protein 2/3 complex subunit 3 | 4.46 | 0.0461 |
| DNAJA1 | DnaJ homolog subfamily A member 1 | 4.18 | 0.0469 |
| FUCA1 | Tissue alpha-L-fucosidase | 3.87 | 0.0487 |

**Supplemental Table S2:** Protein identification, fold difference (FD), and p-values associated with differentially abundant proteins for pooled Female or Male embryos considered normal (Normal) and delayed (Delayed) in timing to reach the expanding blastocyst stage of development. Three replicates with total embryos for Male (n=40) and Female (n=42). Fold difference indicates Female relative to Male.

| **ID** | **Name** | **FD** | **P-value** |
| --- | --- | --- | --- |
| YWHAE | 14-3-3 protein epsilon | 0.47 | 0.001 |
| PRDX4 | Peroxiredoxin-4 | 10.57 | 0.002 |
| GCAT | 2-amino-3-ketobutyrate coenzyme A ligase, mitochondrial | 3.31 | 0.003 |
| NUDT21 | Cleavage and polyadenylation specificity factor subunit 5 | 2.97 | 0.003 |
| FYN | Tyrosine-protein kinase Fyn | 5.66 | 0.004 |
| H1-2 | Histone H1.2 | 0.29 | 0.004 |
| NASP | Nuclear autoantigenic sperm protein | 0.37 | 0.004 |
| SAMM50 | Sorting and assembly machinery component 50 homolog | 6.01 | 0.005 |
| OARD1 | ADP-ribose glycohydrolase OARD1 | 0.25 | 0.006 |
| BPNT1 | 3'(2'),5'-bisphosphate nucleotidase 1 | 0.12 | 0.006 |
| SND1 | Staphylococcal nuclease domain-containing protein 1 | 0.43 | 0.007 |
| TUBA3 | Tubulin alpha-3 chain | 4.71 | 0.007 |
| PSMB7 | Proteasome subunit beta type-7 | 0.10 | 0.010 |
| ATP6V1E1 | V-type proton ATPase subunit E 1 | 0.23 | 0.011 |
| ARPP19 | cAMP-regulated phosphoprotein 19 | 0.29 | 0.014 |
| CLPTM1 | Putative lipid scramblase CLPTM1 | 0.04 | 0.014 |
| COTL1 | Coactosin-like protein | 0.29 | 0.015 |
| RPS3A | Small ribosomal subunit protein eS1 | 0.41 | 0.018 |
| MAPK1 | Mitogen-activated protein kinase 1 | 0.23 | 0.019 |
| SEC13 | Protein SEC13 homolog | 0.28 | 0.020 |
| IDH3B | Isocitrate dehydrogenase [NAD] subunit beta, mitochondrial | 4.02 | 0.021 |
| BCHE | Cholinesterase | 3.44 | 0.024 |
| CLPP | ATP-dependent Clp protease proteolytic subunit, mitochondrial | 0.31 | 0.024 |
| UBE2V1 | Ubiquitin-conjugating enzyme E2 variant 1 | 0.17 | 0.024 |
| ORM1 | Alpha-1-acid glycoprotein | 3.58 | 0.025 |
| POLR2E | DNA-directed RNA polymerases I, II, and III subunit RPABC1 | 0.05 | 0.028 |
| FTH1 | Ferritin heavy chain | 0.55 | 0.029 |
| GET4 | Golgi to ER traffic protein 4 homolog | 0.21 | 0.029 |
| SEPTIN2 | Septin-2 | 1.87 | 0.029 |
| HMBS | Porphobilinogen deaminase | 0.16 | 0.030 |
| CFL2 | Cofilin-2 | 0.29 | 0.031 |
| H1-3 | Histone H1.3 | 0.26 | 0.031 |
| TAF8 | Transcription initiation factor TFIID subunit 8 | 0.11 | 0.031 |
| FKBP4 | Peptidyl-prolyl cis-trans isomerase FKBP4 | 0.37 | 0.032 |
| P2RX4 | P2X purinoceptor 4 | 0.30 | 0.032 |
| KRT10 | Keratin, type I cytoskeletal 10 | 0.61 | 0.032 |
| ATP6V1B1 | V-type proton ATPase subunit B, kidney isoform | 0.19 | 0.034 |
| EIF4A2 | Eukaryotic initiation factor 4A-II | 0.08 | 0.034 |
| ZNF259 | Zinc finger protein ZPR1 | 0.12 | 0.035 |
| PYCR3 | Pyrroline-5-carboxylate reductase 3 | 0.22 | 0.035 |
| HINT1 | Adenosine 5'-monophosphoramidase HINT1 | 0.34 | 0.035 |
| H1-1 | Histone H1.1 | 0.30 | 0.035 |
| ZBTB8OS | Protein archease | 0.25 | 0.036 |
| DDT | D-dopachrome decarboxylase | 2.37 | 0.037 |
| ACP1 | Low molecular weight phosphotyrosine protein phosphatase | 3.68 | 0.038 |
| RAN | GTP-binding nuclear protein Ran | 0.26 | 0.038 |
| HMGCR | 3-hydroxy-3-methylglutaryl-coenzyme A reductase | 0.02 | 0.039 |
| PRMT5 | Protein arginine N-methyltransferase 5 | 0.40 | 0.039 |
| SLC2A1 | Solute carrier family 2, facilitated glucose transporter member 1 | 2.93 | 0.040 |
| EIF4A3 | Eukaryotic initiation factor 4A-III | 0.37 | 0.041 |
| PABPN1 | Polyadenylate-binding protein 2 | 0.19 | 0.041 |
| EHD1 | EH domain-containing protein 1 | 2.30 | 0.043 |
| PIK3R1 | Phosphatidylinositol 3-kinase regulatory subunit alpha | 0.31 | 0.044 |
| MYG1 | MYG1 exonuclease | 0.14 | 0.044 |
| FECH | Ferrochelatase, mitochondrial | 0.18 | 0.044 |
| FADD | FAS-associated death domain protein | 0.17 | 0.045 |
| VNN1 | Pantetheinase | 4.03 | 0.047 |
| WASL | Actin nucleation-promoting factor WASL | 0.28 | 0.047 |
| SERPINA3-1 | Serpin A3-1 | 5.26 | 0.047 |
| PFKL | ATP-dependent 6-phosphofructokinase, liver type | 4.05 | 0.048 |
| SOD2 | Superoxide dismutase [Mn], mitochondrial | 6.42 | 0.049 |
| NDUFB6 | NADH dehydrogenase [ubiquinone] 1 beta subcomplex subunit 6 | 0.12 | 0.049 |
| UCHL1 | Ubiquitin carboxyl-terminal hydrolase isozyme L1 | 2.07 | 0.050 |
| VAMP3 | Vesicle-associated membrane protein 3 | 0.37 | 0.050 |

**Supplemental Table S3:** Protein identification, fold difference (FD), and p-values associated with differentially abundant proteins for Female or Male embryos considered normal (Normal) in timing to reach the expanding blastocyst stage of development. Three replicates with total embryos for Male (n=20) and Female (n=24). Fold difference (FD) indicates Female relative to Male.

| **ID** | **Name** | **FD** | **P-value** |
| --- | --- | --- | --- |
| KRT28 | Keratin, type I cytoskeletal 28 | 0.28 | 0.001 |
| OARD1 | ADP-ribose glycohydrolase OARD1 | 0.24 | 0.001 |
| SLC25A11 | Mitochondrial 2-oxoglutarate/malate carrier protein | 0.03 | 0.001 |
| H1-0 | Histone H1.0 | 0.24 | 0.001 |
| P2RX4 | P2X purinoceptor 4 | 0.24 | 0.002 |
| HMGB2 | High mobility group protein B2 | 0.22 | 0.003 |
| PSMA6 | Proteasome subunit alpha type-6 | 0.07 | 0.003 |
| ANXA11 | Annexin A11 | 5.15 | 0.003 |
| STT3A | Dolichyl-diphosphooligosaccharide--protein glycosyltransferase subunit STT3A | 0.03 | 0.003 |
| H1-1 | Histone H1.1 | 0.21 | 0.004 |
| ATP6V0A1 | V-type proton ATPase subunit a | 0.21 | 0.004 |
| DDX19A | RNA helicase | 3.22 | 0.004 |
| RABEP2 | Rab GTPase-binding effector protein 2 | 3.12 | 0.006 |
| IDI1 | Isopentenyl-diphosphate Delta-isomerase 1 | 2.65 | 0.006 |
| ARF4 | ADP-ribosylation factor 4 | 2.69 | 0.008 |
| VPS4B | Vacuolar protein sorting-associated protein 4B | 0.07 | 0.008 |
| COX6B1 | Cytochrome c oxidase subunit 6B1 | 0.18 | 0.009 |
| RAB6B | Ras-related protein Rab-6B | 0.11 | 0.009 |
| HDAC1 | Histone deacetylase 1 | 0.17 | 0.011 |
| CIAPIN1 | Anamorsin | 0.26 | 0.011 |
| FTH1 | Ferritin heavy chain | 0.07 | 0.011 |
| NDUFB6 | NADH dehydrogenase [ubiquinone] 1 beta subcomplex subunit 6 | 0.17 | 0.013 |
| RTN3 | Reticulon-3 | 0.16 | 0.014 |
| ARPP19 | cAMP-regulated phosphoprotein 19 | 0.05 | 0.015 |
| RPL13 | Large ribosomal subunit protein eL13 | 0.10 | 0.015 |
| PPP1CB | Serine/threonine-protein phosphatase PP1-beta catalytic subunit | 8.75 | 0.015 |
| TMEM30A | Cell cycle control protein 50A | 1.89 | 0.016 |
| AURKA | Aurora kinase A | 0.33 | 0.017 |
| SKP1 | S-phase kinase-associated protein 1 | 3.63 | 0.017 |
| EMC7 | Endoplasmic reticulum membrane protein complex subunit 7 | 0.15 | 0.018 |
| PRKAR1A | cAMP-dependent protein kinase type I-alpha regulatory subunit | 0.15 | 0.018 |
| EIF3J | Eukaryotic translation initiation factor 3 subunit J | 2.22 | 0.018 |
| HDHD2 | Haloacid dehalogenase-like hydrolase domain-containing protein 2 | 0.14 | 0.019 |
| CDC5L | Cell division cycle 5-like protein | 5.88 | 0.020 |
| COL1A1 | Collagen alpha-1(I) chain | 0.03 | 0.025 |
| DYNLL2 | Dynein light chain 2, cytoplasmic | 3.78 | 0.026 |
| VPS29 | Vacuolar protein sorting-associated protein 29 | 1.67 | 0.027 |
| HMBS | Porphobilinogen deaminase | 0.12 | 0.028 |
| ACTN1 | Alpha-actinin-1 | 2.10 | 0.028 |
| SNRPF | Small nuclear ribonucleoprotein F | 6.85 | 0.031 |
| PSMC2 | 26S proteasome regulatory subunit 7 | 1.79 | 0.031 |
| SLC25A6 | ADP/ATP translocase 3 | 0.08 | 0.031 |
| FHIT | Bis(5'-adenosyl)-triphosphatase | 2.17 | 0.033 |
| OGDH | 2-oxoglutarate dehydrogenase complex component E1 | 0.33 | 0.034 |
| CLPP | ATP-dependent Clp protease proteolytic subunit, mitochondrial | 0.07 | 0.035 |
| PSMA1 | Proteasome subunit alpha type-1 | 0.27 | 0.035 |
| CPNE1 | Copine-1 | 5.19 | 0.035 |
| AK4 | Adenylate kinase 4, mitochondrial | 0.09 | 0.035 |
| DDT | D-dopachrome decarboxylase | 2.62 | 0.036 |
| TAF8 | Transcription initiation factor TFIID subunit 8 | 0.10 | 0.036 |
| ALB | Albumin | 3.25 | 0.038 |
| CHTOP | Chromatin target of PRMT1 protein | 0.10 | 0.038 |
| MARCKSL1 | MARCKS-related protein | 0.17 | 0.039 |
| MESD | LRP chaperone MESD | 4.12 | 0.039 |
| VDAC2 | Voltage-dependent anion-selective channel protein 2 | 0.23 | 0.039 |
| ATP6V1C1 | V-type proton ATPase subunit C 1 | 4.52 | 0.040 |
| DENR | Density-regulated protein | 2.22 | 0.041 |
| NAPA | Alpha-soluble NSF attachment protein | 3.58 | 0.042 |
| PSMB3 | Proteasome subunit beta type-3 | 0.00 | 0.042 |
| EIF3B | Eukaryotic translation initiation factor 3 subunit B | 2.85 | 0.043 |
| SFN | 14-3-3 protein sigma | 1.86 | 0.045 |
| SH3GL1 | Endophilin-A2 | 3.30 | 0.045 |
| H2AC20 | Histone H2A type 2-C | 0.11 | 0.045 |
| RNF126 | E3 ubiquitin-protein ligase RNF126 | 0.08 | 0.046 |
| ATXN10 | Ataxin-10 | 3.31 | 0.050 |

**Supplemental Table S4:** Protein identification, fold difference (FD), and p-values associated with differentially abundant proteins for pooled Male embryos considered Normal and Delayed in developmental timing. Three replicates with total embryos for Normal (n=20) and Delayed (n=20). Fold difference indicates Male Delayed relative to Male Normal.

| **ID** | **Name** | **FD** | **P-value** |
| --- | --- | --- | --- |
| TGM2 | Protein-glutamine gamma-glutamyltransferase 2 | 0.12 | 0.002 |
| RPL13A | Large ribosomal subunit protein uL13 | 0.18 | 0.003 |
| MFGE8 | Lactadherin | 0.20 | 0.011 |
| RPL35 | Large ribosomal subunit protein uL29 | 0.11 | 0.018 |
| PRPS1 | Ribose-phosphate pyrophosphokinase 1 | 0.08 | 0.019 |
| NARS | Asparagine--tRNA ligase, cytoplasmic | 0.08 | 0.021 |
| ATP5IF1 | ATPase inhibitor, mitochondrial | 0.02 | 0.022 |
| ZP4 | Zona pellucida sperm-binding protein 4 | 0.37 | 0.026 |
| MVP | Major vault protein | 0.38 | 0.029 |
| HSPB1 | Heat shock protein beta-1 | 0.29 | 0.030 |
| PLS1 | Plastin-1 | 4.61 | 0.034 |
| CIAPIN1 | Anamorsin | 0.12 | 0.034 |
| RPL10A | Large ribosomal subunit protein uL1 | 0.15 | 0.040 |
| RPL18 | Large ribosomal subunit protein eL18 | 0.15 | 0.042 |
| PUF60 | Poly(U)-binding-splicing factor PUF60 | 0.18 | 0.044 |
| RPS7 | Small ribosomal subunit protein eS7 | 0.16 | 0.045 |
| AHCY | Adenosylhomocysteinase | 0.09 | 0.046 |
| NSDHL | Sterol-4-alpha-carboxylate 3-dehydrogenase, decarboxylating | 5.27 | 0.049 |

**Supplemental Table S5:** Protein identification, fold difference (FD), and P-values associated with differentially abundant proteins for pooled Female embryos considered Normal and Delayed in timing to reach the expanding blastocyst stage of development. Three replicates with total embryos for Normal (n=24) and Delayed (n=18). Fold difference indicates Female Delayed relative to Female Normal.

| **ID** | **Name** | **FD** | **P-value** |
| --- | --- | --- | --- |
| TKT | Transketolase | 0.44 | 0.0001 |
| DNM1L | Dynamin-1-like protein | 0.19 | 0.0006 |
| LAMP1 | Lysosomal membrane glycoprotein 1 | 0.27 | 0.0008 |
| EEF1A1 | Elongation factor 1-alpha 1 | 3.00 | 0.0009 |
| CLINT1 | Clathrin interactor 1 | 0.45 | 0.0010 |
| NUDC | Nuclear migration protein | 0.33 | 0.0012 |
| NPM1 | Nucleophosmin | 0.37 | 0.0012 |
| RPL17 | Large ribosomal subunit protein uL22 | 0.46 | 0.0013 |
| ARF4 | ADP-ribosylation factor 4 | 0.33 | 0.0013 |
| EIF3J | Eukaryotic translation initiation factor 3 subunit J | 0.15 | 0.0015 |
| IDI1 | Isopentenyl-diphosphate Delta-isomerase 1 | 0.15 | 0.0020 |
| NAP1L4 | Nucleosome assembly protein 1-like 4 | 0.35 | 0.0029 |
| TMSB4 | Thymosin beta-4 | 0.23 | 0.0030 |
| HNRNPK | Heterogeneous nuclear ribonucleoprotein K | 0.39 | 0.0030 |
| CS | Citrate synthase | 0.51 | 0.0033 |
| RPL3 | Large ribosomal subunit protein uL3 | 0.27 | 0.0042 |
| RANBP1 | Ran-specific GTPase-activating protein | 0.49 | 0.0042 |
| F11R | Junctional adhesion molecule A | 0.33 | 0.0044 |
| ERLIN2 | Erlin-2 | 0.50 | 0.0044 |
| DDX1 | ATP-dependent RNA helicase DDX1 | 0.34 | 0.0049 |
| HDGF | Hepatoma-derived growth factor | 0.02 | 0.0054 |
| PCMT1 | Protein-L-isoaspartate(D-aspartate) O-methyltransferase | 0.42 | 0.0060 |
| ATP6V1C1 | V-type proton ATPase subunit C 1 | 0.21 | 0.0063 |
| RPS18 | Small ribosomal subunit protein uS13 | 0.34 | 0.0067 |
| PFN2 | Profilin-2 | 0.42 | 0.0074 |
| KRT14 | Keratin, type I cytoskeletal 14 | 18.77 | 0.0086 |
| SMU1 | WD40 repeat-containing protein SMU1 | 0.07 | 0.0087 |
| YWHAG | 14-3-3 protein gamma | 0.39 | 0.0088 |
| EIF3G | Eukaryotic translation initiation factor 3 subunit G | 0.40 | 0.0089 |
| SRSF3 | Serine/arginine-rich splicing factor 3 | 0.16 | 0.0094 |
| PSMC5 | 26S proteasome regulatory subunit 8 | 0.41 | 0.0103 |
| SNX5 | Sorting nexin-5 | 0.27 | 0.0104 |
| RPL7 | Large ribosomal subunit protein uL30 | 16.88 | 0.0106 |
| PRKAR2A | cAMP-dependent protein kinase type II-alpha regulatory subunit | 0.31 | 0.0108 |
| ATXN10 | Ataxin-10 | 0.08 | 0.0111 |
| PARP1 | Poly [ADP-ribose] polymerase 1 | 0.56 | 0.0111 |
| ISOC1 | Isochorismatase domain-containing protein 1 | 2.48 | 0.0120 |
| MCM3 | DNA replication licensing factor Mcm3 | 0.33 | 0.0120 |
| SPCS1 | Signal peptidase complex subunit 1 | 2.49 | 0.0120 |
| CSTF2 | Cleavage stimulation factor subunit 2 | 2.45 | 0.0120 |
| RPL35A | RPL35A ribosomal protein L35a | 0.19 | 0.0120 |
| FN1 | Fibronectin | 1.98 | 0.0120 |
| ARPC3 | Actin-related protein 2/3 complex subunit 3 | 2.44 | 0.0121 |
| ADSS2 | Adenylosuccinate synthetase isozyme 2 | 2.52 | 0.0121 |
| PARL | Presenilin-associated rhomboid-like protein, mitochondrial | 2.52 | 0.0121 |
| RPS23 | Small ribosomal subunit protein uS12 | 2.52 | 0.0121 |
| MMAB | Corrinoid adenosyltransferase MMAB | 2.54 | 0.0122 |
| MGST3 | Glutathione S-transferase 3, mitochondrial | 2.54 | 0.0122 |
| DYNLL2 | Dynein light chain 2, cytoplasmic | 0.13 | 0.0122 |
| SKP1 | S-phase kinase-associated protein 1 | 0.19 | 0.0124 |
| SSB | Single-stranded DNA-binding protein | 0.36 | 0.0124 |
| CTH | Cystathionine gamma-lyase | 2.57 | 0.0124 |
| ARPC4 | Actin-related protein 2/3 complex subunit 4 | 2.57 | 0.0125 |
| DBI | Acyl-CoA-binding protein | 2.39 | 0.0126 |
| STT3A | Dolichyl-diphosphooligosaccharide--protein glycosyltransferase subunit STT3A | 2.59 | 0.0127 |
| CDC37 | Hsp90 co-chaperone Cdc37 | 0.30 | 0.0128 |
| PSMB1 | Proteasome subunit beta type-1 | 2.63 | 0.0131 |
| PCNA | Proliferating cell nuclear antigen | 0.32 | 0.0132 |
| SCIN | Scinderin | 2.65 | 0.0135 |
| PFDN2 | Prefoldin subunit 2 | 2.69 | 0.0140 |
| RBMX | RNA-binding motif protein, X chromosome | 0.09 | 0.0146 |
| HSP90AA1 | Heat shock protein HSP 90-alpha | 0.39 | 0.0150 |
| GPX4 | Phospholipid hydroperoxide glutathione peroxidase | 2.77 | 0.0154 |
| SUCLA2 | Succinate--CoA ligase | 0.26 | 0.0155 |
| SDHB | Succinate dehydrogenase [ubiquinone] iron-sulfur subunit, mitochondrial | 0.23 | 0.0159 |
| RAB3C | Ras-related protein Rab-3C | 2.81 | 0.0162 |
| TUBA3 | Tubulin alpha-3 chain | 0.29 | 0.0163 |
| MVP | Major vault protein | 0.39 | 0.0165 |
| UBE2L3 | Ubiquitin-conjugating enzyme E2 L3 | 0.22 | 0.0166 |
| RAB18 | RAB18 | 2.84 | 0.0168 |
| RPS25 | Small ribosomal subunit protein eS25 | 2.26 | 0.0171 |
| TXNRD1 | Thioredoxin reductase 1, cytoplasmic | 2.87 | 0.0174 |
| RENBP | N-acylglucosamine 2-epimerase | 0.36 | 0.0178 |
| GGPS1 | Geranylgeranyl pyrophosphate synthase | 0.13 | 0.0179 |
| TMEM30A | Cell cycle control protein 50A | 0.40 | 0.0181 |
| MTHFD1L | Monofunctional C1-tetrahydrofolate synthase, mitochondrial | 0.48 | 0.0183 |
| RPL24 | Large ribosomal subunit protein eL24 | 45.10 | 0.0185 |
| FUCA1 | Tissue alpha-L-fucosidase | 2.24 | 0.0185 |
| RPL30 | Large ribosomal subunit protein eL30 | 0.44 | 0.0194 |
| FAU | Small ribosomal subunit protein eS30 | 2.97 | 0.0197 |
| GRPEL1 | GrpE protein homolog 1, mitochondrial | 0.40 | 0.0197 |
| PPP1CA | Serine/threonine-protein phosphatase PP1-alpha catalytic subunit | 2.22 | 0.0200 |
| PRDX4 | Peroxiredoxin-4 | 5.26 | 0.0203 |
| MAL2 | Protein MAL2 | 2.21 | 0.0203 |
| LGALS1 | Galectin-1 | 1.99 | 0.0205 |
| ATP5MF | ATP synthase subunit f, mitochondrial | 2.21 | 0.0206 |
| PHPT1 | 14 kDa phosphohistidine phosphatase | 0.14 | 0.0207 |
| SMS | Somatostatin | 0.43 | 0.0210 |
| CLTA | Clathrin light chain A | 0.23 | 0.0212 |
| DLD | Dihydrolipoyl dehydrogenase, mitochondrial | 0.44 | 0.0222 |
| FKBP1A | Peptidyl-prolyl cis-trans isomerase FKBP1A | 0.28 | 0.0229 |
| STK25 | Serine/threonine-protein kinase 25 | 2.18 | 0.0237 |
| TPP1 | Tripeptidyl-peptidase 1 | 2.18 | 0.0244 |
| KRT78 | Keratin, type II cytoskeletal 78 | 4.59 | 0.0245 |
| MSN | Moesin | 0.37 | 0.0249 |
| TECR | Very-long-chain enoyl-CoA reductase | 2.17 | 0.0250 |
| RAD23B | UV excision repair protein RAD23 homolog B | 0.24 | 0.0251 |
| RPL26 | E3 UFM1-protein ligase 1 | 0.31 | 0.0252 |
| DAG1 | Dystroglycan 1 | 0.06 | 0.0254 |
| SEPTIN7 | Septin-7 | 0.28 | 0.0260 |
| HDDC2 | 5'-deoxynucleotidase HDDC2 | 125.05 | 0.0261 |
| SNRPD2 | Small nuclear ribonucleoprotein Sm D2 | 0.14 | 0.0263 |
| HINT1 | Adenosine 5'-monophosphoramidase HINT1 | 2.16 | 0.0265 |
| DHFR | Dihydrofolate reductase | 3.25 | 0.0267 |
| NAPA | Alpha-soluble NSF attachment protein | 0.15 | 0.0270 |
| DLAT | Dihydrolipoyllysine-residue acetyltransferase component of pyruvate dehydrogenase complex | 4.22 | 0.0276 |
| PFDN4 | Prefoldin subunit 4 | 0.14 | 0.0279 |
| MFGE8 | Lactadherin | 2.70 | 0.0285 |
| VPS35 | Vacuolar protein sorting-associated protein 35 | 0.39 | 0.0289 |
| HDAC1 | Histone deacetylase 1 | 2.15 | 0.0295 |
| RSL1D1 | Ribosomal L1 domain-containing protein 1 | 0.22 | 0.0295 |
| EIF5A | Eukaryotic translation initiation factor 5A-1 | 0.21 | 0.0295 |
| LTF | Lactotransferrin | 3.37 | 0.0297 |
| EEF1B | Elongation factor 1-beta | 0.30 | 0.0321 |
| KRTCAP2 | Keratinocyte-associated protein 2 | 2.35 | 0.0321 |
| ATP6V1D | V-type proton ATPase subunit D | 2.38 | 0.0322 |
| ARPC1A | Actin-related protein 2/3 complex subunit 1A | 2.38 | 0.0322 |
| PSMA6 | Proteasome subunit alpha type-6 | 2.39 | 0.0323 |
| PCYT2 | Ethanolamine-phosphate cytidylyltransferase | 2.40 | 0.0323 |
| PLD3 | 5'-3' exonuclease PLD3 | 3.48 | 0.0324 |
| YARS1 | Tyrosine--tRNA ligase, cytoplasmic | 0.32 | 0.0324 |
| NIFK | MKI67 FHA domain-interacting nucleolar phosphoprotein | 2.13 | 0.0327 |
| NDUFA11 | NADH dehydrogenase [ubiquinone] 1 alpha subcomplex subunit 11 | 2.44 | 0.0328 |
| PTRH2 | Peptidyl-tRNA hydrolase 2, mitochondrial | 2.26 | 0.0329 |
| ASAH1 | Acid ceramidase | 2.45 | 0.0329 |
| SNRPD1 | Small nuclear ribonucleoprotein Sm D1 | 2.26 | 0.0330 |
| VDAC3 | Voltage-dependent anion-selective channel protein 3 | 3.89 | 0.0336 |
| RTN3 | Reticulon-3 | 2.23 | 0.0337 |
| UQCR10 | Cytochrome b-c1 complex subunit 9 | 2.12 | 0.0339 |
| TRMT10C | tRNA methyltransferase 10 homolog C | 0.09 | 0.0340 |
| RABAC1 | Prenylated Rab acceptor protein 1 | 2.22 | 0.0341 |
| NDUFAF4 | NADH dehydrogenase [ubiquinone] 1 alpha subcomplex assembly factor 4 | 2.22 | 0.0341 |
| MT-ND5 | NADH-ubiquinone oxidoreductase chain 5 | 2.53 | 0.0344 |
| COL1A2 | Collagen alpha-2(I) chain | 2.53 | 0.0345 |
| AK4 | Adenylate kinase 4, mitochondrial | 6.34 | 0.0345 |
| MVD | Diphosphomevalonate decarboxylase | 2.20 | 0.0347 |
| TIMM21 | Mitochondrial import inner membrane translocase subunit Tim21 | 0.17 | 0.0356 |
| SPNS1 | Protein spinster homolog 1 | 0.15 | 0.0357 |
| UQCRB | Cytochrome b-c1 complex subunit 7 | 0.31 | 0.0360 |
| CPT2 | Carnitine O-palmitoyltransferase 2, mitochondrial | 0.20 | 0.0360 |
| ACTL6B | Actin-like protein 6B | 2.62 | 0.0365 |
| DDX19A | ATP-dependent RNA helicase DDX19A | 0.22 | 0.0365 |
| SNRPF | Small nuclear ribonucleoprotein F | 0.09 | 0.0369 |
| VPS28 | Vacuolar protein sorting-associated protein 28 homolog | 0.07 | 0.0369 |
| CLTB | Clathrin light chain B | 0.19 | 0.0372 |
| ATP5PD | ATP synthase subunit d, mitochondrial | 0.58 | 0.0372 |
| RPS7 | Small ribosomal subunit protein eS7 | 0.36 | 0.0374 |
| STOML2 | Stomatin-like protein 2, mitochondrial | 0.38 | 0.0375 |
| YWHAZ | 14-3-3 protein zeta/delta | 0.22 | 0.0380 |
| GNS | N-acetylglucosamine-6-sulfatase | 0.45 | 0.0380 |
| COX7A2L | Cytochrome c oxidase subunit 7A-related protein, mitochondrial | 2.69 | 0.0385 |
| KGD4 | Alpha-ketoglutarate dehydrogenase component 4 | 2.70 | 0.0386 |
| DARS1 | Aspartate--tRNA ligase, cytoplasmic | 1.56 | 0.0388 |
| HSP90AB1 | Heat shock protein HSP 90-beta | 0.58 | 0.0390 |
| SLC25A11 | Mitochondrial 2-oxoglutarate/malate carrier protein | 3.78 | 0.0396 |
| DSC2 | Desmocollin-2 | 3.79 | 0.0399 |
| SHMT2 | Serine hydroxymethyltransferase, mitochondrial | 2.76 | 0.0403 |
| GBA1 | Lysosomal acid glucosylceramidase | 2.11 | 0.0411 |
| CLIC1 | Chloride intracellular channel protein 1 | 0.48 | 0.0418 |
| PSMC6 | 26S proteasome regulatory subunit 10B | 2.82 | 0.0422 |
| MRPS25 | Small ribosomal subunit protein mS25 | 2.09 | 0.0426 |
| ACO1 | Aconitate hydratase 1 | 2.10 | 0.0427 |
| APMAP | Adipocyte plasma membrane-associated protein | 2.85 | 0.0430 |
| CPNE1 | Copine-1 | 0.25 | 0.0431 |
| MSH2 | DNA mismatch repair protein Msh2 | 0.21 | 0.0431 |
| NUP93 | Nuclear pore complex protein Nup93 | 0.20 | 0.0432 |
| DNAJA1 | DnaJ homolog subfamily A member 1 | 2.09 | 0.0436 |
| SH3GL1 | Endophilin-A2 | 0.16 | 0.0438 |
| ARHGAP29 | Rho GTPase-activating protein 29 | 2.08 | 0.0446 |
| AP2S1 | AP complex subunit sigma | 2.91 | 0.0450 |
| RMDN1 | Regulator of microtubule dynamics protein 1 | 0.19 | 0.0452 |
| TMX1 | Thioredoxin-related transmembrane protein 1 | 9.47 | 0.0453 |
| SAE1 | SUMO-activating enzyme subunit 1 | 0.20 | 0.0465 |
| AHSG | Alpha-2-HS-glycoprotein | 2.99 | 0.0474 |
| VDAC2 | Voltage-dependent anion-selective channel protein 2 | 2.77 | 0.0474 |
| NASP | Nuclear autoantigenic sperm protein | 0.45 | 0.0475 |
| TGM2 | Protein-glutamine gamma-glutamyltransferase 2 | 0.16 | 0.0476 |
| MIF | Macrophage migration inhibitory factor | 3.00 | 0.0477 |
| EIF2S2 | Eukaryotic translation initiation factor 2 subunit 1 | 0.26 | 0.0478 |
| RER1 | Protein RER1 | 0.36 | 0.0487 |
| PEBP1 | Phosphatidylethanolamine-binding protein 1 | 0.38 | 0.0495 |
| BCS1L | Mitochondrial chaperone BCS1 | 0.30 | 0.0496 |

**Supplemental Table S6:** Protein identification, overall p-values, and significant group differences associated with differentially abundant proteins for Male Normal (MN), Female Normal (FN), Male Delayed (MD) and Female Delayed (FD). Three replicates with total embryos for MN (n=20), FN (n=24), MD (n=20) and FD (n=18). P-value was determined by one-way ANOVA, and Fisher’s LSD indicates significant (p<0.05) comparisons among individual groups with higher protein abundance in first group relative to second group.

| **ID** | **Name** | **F-stat** | **P-value** | **Fisher's LSD** |
| --- | --- | --- | --- | --- |
| PRDX4 | Peroxiredoxin-4 | 20.8 | 0.0004 | Female, delayed - Female, normal; Female, delayed - Male, delayed; Female, delayed - Male, normal; Female, normal - Male, delayed; Female, normal - Male, normal |
| ARPP19 | cAMP-regulated phosphoprotein 19 | 14.3 | 0.0014 | Female, delayed - Female, normal; Male, delayed - Female, normal; Male, normal - Female, normal |
| MFGE8 | Lactadherin | 11.9 | 0.0025 | Female, delayed - Female, normal; Female, delayed - Male, delayed; Male, normal - Female, normal; Male, normal - Male, delayed |
| YWHAE | 14-3-3 protein epsilon | 11.2 | 0.0031 | Male, delayed - Female, delayed; Male, normal - Female, delayed; Male, delayed - Female, normal; Male, normal - Female, normal |
| TGM2 | Protein-glutamine gamma-glutamyltransferase 2 | 9.3 | 0.0055 | Female, normal - Female, delayed; Male, normal - Female, delayed; Female, normal - Male, delayed; Male, normal - Male, delayed |
| NASP | Nuclear autoantigenic sperm protein | 9.2 | 0.0056 | Female, normal - Female, delayed; Male, delayed - Female, delayed; Male, normal - Female, delayed |
| MVP | Major vault protein | 8.6 | 0.0069 | Female, normal - Female, delayed; Male, normal - Female, delayed; Female, normal - Male, delayed; Male, normal - Male, delayed |
| PRPS1 | Ribose-phosphate pyrophosphokinase 1 | 8.5 | 0.0073 | Female, delayed - Male, delayed; Female, normal - Male, delayed; Male, normal - Male, delayed |
| TUBA3 | Tubulin alpha-3 chain | 8.2 | 0.0081 | Female, normal - Female, delayed; Female, normal - Male, delayed; Female, normal - Male, normal |
| HINT1 | Adenosine 5'-monophosphoramidase HINT1 | 7.1 | 0.0122 | Male, delayed - Female, delayed; Male, delayed - Female, normal; Male, delayed - Male, normal |
| ATP5MF | ATP synthase subunit f, mitochondrial | 7.0 | 0.0124 | Female, delayed - Female, normal; Female, delayed - Male, delayed; Female, delayed - Male, normal |
| UCHL1 | Ubiquitin carboxyl-terminal hydrolase isozyme L1 | 6.9 | 0.0132 | Female, delayed - Male, delayed; Female, normal - Male, delayed; Male, normal - Male, delayed |
| CIAPIN1 | Anamorsin | 6.7 | 0.0141 | Female, normal - Female, delayed; Male, normal - Female, delayed; Male, normal - Male, delayed |
| VDAC2 | Voltage-dependent anion-selective channel protein 2 | 6.5 | 0.0155 | Female, delayed - Female, normal; Male, delayed - Female, normal; Male, normal - Female, normal |
| BLVRB | Flavin reductase (NADPH) | 6.4 | 0.0164 | Female, delayed - Female, normal; Female, delayed - Male, delayed; Female, delayed - Male, normal |
| COTL1 | Coactosin-like protein | 6.4 | 0.0164 | Female, normal - Female, delayed; Male, delayed - Female, delayed; Male, normal - Female, delayed |
| RPS25 | Small ribosomal subunit protein eS25 | 6.2 | 0.0174 | Female, delayed - Female, normal; Female, delayed - Male, delayed |
| BCHE | Cholinesterase | 6.1 | 0.0184 | Female, delayed - Female, normal; Female, delayed - Male, delayed; Female, delayed - Male, normal |
| EEF1A1 | Elongation factor 1-alpha 1 | 5.8 | 0.0214 | Female, delayed - Female, normal; Male, normal - Female, normal |
| RPS7 | Small ribosomal subunit protein eS7 | 5.7 | 0.0219 | Male, normal - Female, delayed; Female, normal - Male, delayed; Male, normal - Male, delayed |
| LAMP1 | Lysosome-associated membrane glycoprotein 1 | 5.5 | 0.0240 | Female, normal - Female, delayed; Male, normal - Female, delayed |
| VDAC3 | Voltage-dependent anion-selective channel protein 3 | 5.4 | 0.0253 | Female, delayed - Female, normal; Male, delayed - Female, normal; Male, normal - Female, normal |
| NARS | Asparagine--tRNA ligase, cytoplasmic | 5.4 | 0.0256 | Female, delayed - Male, delayed; Male, normal - Female, normal; Male, normal - Male, delayed |
| KRT14 | Keratin, type I cytoskeletal 14 | 5.2 | 0.0274 | Female, delayed - Female, normal; Female, delayed - Male, delayed; Female, delayed - Male, normal |
| NAPA | Alpha-soluble NSF attachment protein | 5.0 | 0.0300 | Female, normal - Female, delayed; Female, normal - Male, normal |
| FTH1 | Ferritin heavy chain | 5.0 | 0.0301 | Female, delayed - Female, normal; Male, delayed - Female, normal; Male, normal - Female, normal |
| POLR2E | DNA-directed RNA polymerases I, II, and III subunit RPABC1 | 5.0 | 0.0307 | Male, delayed - Female, delayed; Male, delayed - Female, normal |
| FAU | Ubiquitin-like protein FUBI | 4.9 | 0.0321 | Female, delayed - Female, normal; Female, delayed - Male, delayed; Female, delayed - Male, normal |
| RPL7 | Large ribosomal subunit protein uL30 | 4.8 | 0.0334 | Female, delayed - Female, normal; Female, delayed - Male, delayed |
| PFDN2 | Prefoldin subunit 2 | 4.7 | 0.0350 | Female, delayed - Female, normal; Female, delayed - Male, delayed; Female, delayed - Male, normal |
| MT-ND5 | NADH-ubiquinone oxidoreductase chain 5 | 4.7 | 0.0353 | Female, delayed - Female, normal; Female, delayed - Male, delayed; Female, delayed - Male, normal |
| SND1 | Staphylococcal nuclease domain-containing protein 1 | 4.7 | 0.0354 | Male, delayed - Female, delayed; Male, normal - Female, delayed |
| ATP6V1E1 | V-type proton ATPase subunit E 1 | 4.7 | 0.0358 | Male, delayed - Female, delayed; Male, delayed - Female, normal |
| VMP1 | Vacuole membrane protein 1 | 4.5 | 0.0385 | Female, delayed - Female, normal; Female, delayed - Male, delayed |
| CPT2 | Carnitine O-palmitoyltransferase 2, mitochondrial | 4.5 | 0.0388 | Female, normal - Female, delayed; Male, delayed - Female, delayed; Male, normal - Female, delayed |
| SOD2 | Superoxide dismutase [Mn], mitochondrial | 4.5 | 0.0392 | Female, delayed - Female, normal; Female, delayed - Male, delayed; Female, delayed - Male, normal |
| MGST3 | Glutathione S-transferase 3, mitochondrial | 4.5 | 0.0402 | Female, delayed - Female, normal; Female, delayed - Male, delayed; Female, delayed - Male, normal |
| MMAB | Corrinoid adenosyltransferase MMAB | 4.5 | 0.0402 | Female, delayed - Female, normal; Female, delayed - Male, delayed; Female, delayed - Male, normal |
| FYN | Tyrosine-protein kinase Fyn | 4.4 | 0.0406 | Female, delayed - Male, delayed; Female, delayed - Male, normal |
| BPNT1 | 3'(2'),5'-bisphosphate nucleotidase 1 | 4.4 | 0.0411 | Male, delayed - Female, delayed; Male, normal - Female, delayed; Male, delayed - Female, normal |
| RPSA | Small ribosomal subunit protein uS2 | 4.4 | 0.0423 | Female, delayed - Male, delayed; Male, normal - Male, delayed |
| APOD | Apolipoprotein D | 4.3 | 0.0433 | Male, delayed - Female, delayed; Male, delayed - Female, normal; Male, delayed - Male, normal |
| SERPINA3-1 | Serpin A3-1 | 4.3 | 0.0445 | Female, delayed - Female, normal; Female, delayed - Male, delayed; Female, delayed - Male, normal |
| CSTF2 | Cleavage stimulation factor subunit 2 | 4.2 | 0.0456 | Female, delayed - Female, normal; Female, delayed - Male, delayed; Female, delayed - Male, normal |
| ACP1 | Low molecular weight phosphotyrosine protein phosphatase | 4.2 | 0.0462 | Female, delayed - Male, delayed; Female, delayed - Male, normal |
| SAMM50 | Sorting and assembly machinery component 50 homolog | 4.2 | 0.0464 | Female, delayed - Male, delayed; Female, delayed - Male, normal |
| GCAT | 2-amino-3-ketobutyrate coenzyme A ligase, mitochondrial | 4.1 | 0.0478 | Female, delayed - Male, delayed; Female, delayed - Male, normal; Female, normal - Male, delayed; Female, normal - Male, normal |
| VDAC1 | Voltage-dependent anion-selective channel protein 1 | 4.1 | 0.0481 | Female, delayed - Male, delayed; Male, normal - Male, delayed |
